# A multi-task and explainable swin transformer framework for cross-scale computational pathology in gastrointestinal cancer

**DOI:** 10.3389/fonc.2026.1749675

**Published:** 2026-04-21

**Authors:** Qing-Chun Feng, Ting Yang, Hai-Long Guo, Xiao-Yun Wang

**Affiliations:** Shanghai Key Laboratory for Cancer Systems Regulation and Clinical Translation, Department of General Surgery, Jiading District Central Hospital Affiliated Shanghai University of Medicine & Health Sciences, Shanghai, China

**Keywords:** cross-scale recognition, deep learning, digital pathology, explainable artificial intelligence, gastrointestinal cancer, model generalization, multi-task learning, transformer network

## Abstract

**Background:**

Accurate identification and segmentation of tumor and microenvironment features in gastrointestinal cancer (GC) pathology images are crucial for diagnosis, yet challenging for traditional methods. This study aims to develop and validate a deep learning (ML) framework integrating multi-task learning and interpretability mechanisms for cross-scale automatic classification and segmentation of tumor and microenvironmental structures in gastrointestinal cancer histopathological patches, and to evaluate its robustness, output consistency, and decision transparency under controlled benchmark settings.

**Methods:**

We constructed a multi-task learning (MTL) model integrating Swin Transformer, DeepLabV3+, and R2U-Net for joint classification and segmentation. The model was trained and validated on approximately 99,000 H&E-stained images from the GasHisSDB and GCHTID datasets. Preprocessing included color normalization and quality control. Performance was evaluated via five-fold cross-validation. Explainability was assessed using Grad-CAM, Score-CAM, and Layer-wise Relevance Propagation (LRP), with validation from pathology experts.

**Results:**

The multi-task model achieved a classification F1-score of 0.938 ± 0.007 and a segmentation Dice coefficient of 0.839 ± 0.009 on the test set. Compared with ResNet-50, Swin-T obtained higher classification performance, with improved F1-score (0.945 vs. 0.917) and AUC (0.965 vs. 0.907). For small-volume tissues, the LYM Dice reached 0.781. In cross-domain transfer from GCHTID to GasHisSDB, the model achieved an F1-score of 0.902. Under staining perturbations, the Dice decreased by only 2.4%, and Grad-CAM correlation reached r = 0.86. The expert-model agreement rate (EMAR) was 0.864, with a Cohen’s κ of 0.79.

**Conclusion:**

The proposed cross-scale multi-task Transformer framework achieves high-precision recognition and multi-component segmentation of gastrointestinal cancer histopathological images, demonstrating stability and interpretability across scale variations, cross-dataset evaluations, and staining perturbation tests. Overall, this study emphasizes the establishment and validation of a methodological paradigm integrating multi-task joint learning, cross-scale generalization assessment, and interpretable evidence review. As the current validation was conducted under controlled benchmark conditions using strongly annotated patch-level data, the framework should be regarded as a clinically relevant, preclinical validation system. Its feasibility for routine clinical implementation requires further verification through large-scale whole-slide image (WSI) cohorts, prospective multicenter studies, and workflow integration assessments.

## Graphical Abstract

**Figure:**
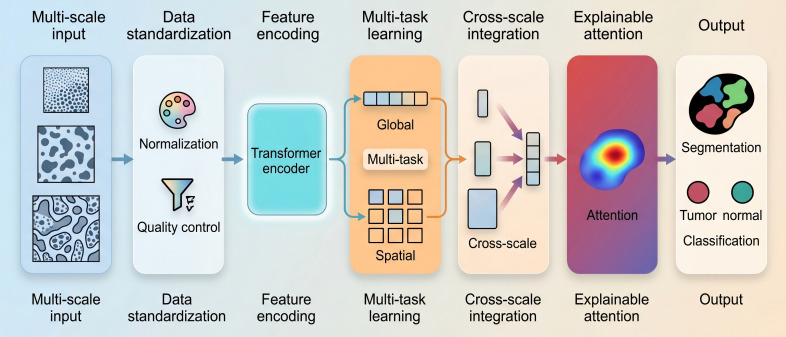
Schematic illustration of the cross-scale automatic recognition mechanism for GC pathological images based on a multi-task Transformer.

## Introduction

Gastrointestinal cancer (GC), including gastric cancer and colorectal cancer, represents one of the leading malignant tumors globally, with incidence and mortality rates continuing to rise, posing a serious threat to human health (1, 2). Statistics indicate that the annual number of new cases of gastric and colorectal cancer has exceeded 4 million, accounting for approximately one-fourth of the global cancer burden, with developing countries contributing nearly 60% of these cases (3). Despite advancements in surgical techniques, radiotherapy, chemotherapy, and targeted therapies, the five-year survival rate for patients with advanced GC remains below 30%. These tumors exhibit significant histological heterogeneity, with distinct subtypes demonstrating marked differences in molecular pathways, immune microenvironments, and clinical phenotypes, which pose considerable challenges for accurate diagnosis and classification (4). Currently, the diagnosis of GC primarily relies on pathological examinations, particularly manual interpretation of hematoxylin and eosin (H&E)-stained slides. However, traditional diagnostic approaches are heavily dependent on pathologists’ expertise and are characterized by high subjectivity, poor reproducibility, and low efficiency. These limitations become particularly pronounced when dealing with large-scale pathological imaging data or complex microenvironmental structures, where manual identification struggles to achieve sufficient accuracy and consistency (5).

With the advancement of whole slide imaging (WSI) technology, traditional glass slide pathology has undergone a digital transformation, laying the technical foundation for artificial intelligence (AI)-based pathological image analysis (6). Digital pathology (DP) enables the storage, sharing, and large-scale analysis of high-resolution images, thereby creating favorable conditions for the application of computer vision algorithms in pathological diagnosis (7). Representative studies have validated that weakly supervised deep learning on large-scale WSIs can achieve clinical-grade diagnostic support and demonstrate feasible deployment pathways within real-world workflows (8). Meanwhile, authoritative reviews have emphasized that staining and scanning variability, inter-center distribution shifts, as well as interpretability and clinical validation procedures, are critical factors affecting the objectivity and generalizability of digital pathology AI systems, and should be systematically addressed in study design (9). For WSI-level tasks with only weak slide-level annotations, Multiple Instance Learning (MIL) has become the mainstream framework. Iterative MIL, key-instance-driven MIL, task-specific multi-level feature learning, and hierarchical deep MIL approaches have continuously advanced both classification performance and interpretability in WSI analysis (10–13). In addition, local blurring caused by out-of-focus scanning can impair tissue detail representation. Recent studies have proposed digital pathology deblurring methods based on local focus quality assessment to enhance image quality and support downstream analytical tasks (14). Deep learning (DL) methods, particularly convolutional neural networks (CNNs), have achieved groundbreaking progress in tasks such as tumor identification, lesion detection, and subtype prediction. These methods have been widely applied to the intelligent diagnosis of pathological images in breast cancer, lung cancer, cervical cancer, and other malignancies (15, 16). In the field of GC, researchers have utilized AI models to identify tumor regions, predict differentiation grades, or detect invasive boundaries. Some models have demonstrated diagnostic performance comparable to that of expert pathologists (17). However, existing studies predominantly focus on single-task problems such as classification or detection. They lack integrated modeling strategies for complex pathological tasks like multi-tissue structure segmentation or tumor microenvironment component identification (18). Moreover, most models are trained and validated on single-center datasets and often fail to achieve cross-center generalization and multi-scale adaptability. This limitation undermines their transferability and stability in clinical applications.

The complexity of gastrointestinal pathological images arises not only from the heterogeneity of diseases but also from multidimensional variations in image scale, tissue types, staining intensity, and slice quality. Image resolutions from different data sources range from 80×80 to 224×224, while various tissue components such as tumor epithelium, lymphocytes, smooth muscle, and cancer-associated stroma exhibit significant variability in morphology and distribution (19). This cross-scale and cross-tissue complexity often leads to information loss or overfitting in traditional DL models during feature representation. Additionally, discrepancies in clinical equipment and stain variation further exacerbate the instability of model performance (20). Although existing studies have explored techniques such as transfer learning and domain adaptation to enhance model robustness, most approaches require additional target domain samples or fine-tuning processes, making true cross-domain generalization challenging (21). More critically, the majority of current AI models rely on “black-box” reasoning without transparent explainable artificial intelligence (XAI) mechanisms. This lack of interpretability prevents clinicians from understanding the basis of model decisions, hindering trust and adoption in practical diagnostic workflows.

In recent years, MTL has been demonstrated as an effective strategy to enhance the performance and generalization of models in medical imaging. By sharing a feature encoder and simultaneously learning tasks such as classification and segmentation, MTL enables models to retain semantic information while improving sensitivity to local structures (22). In the field of pathological imaging, this approach facilitates the integration of tumor identification with tissue component annotation, thereby avoiding information fragmentation and enhancing diagnostic consistency in complex scenarios. Meanwhile, Transformer networks, particularly Swin Transformer (Swin-T), have gained widespread application in medical imaging segmentation and classification tasks due to their GSA mechanism and multi-scale feature extraction capabilities. These networks have also exhibited superior performance in multimodal fusion (23, 24). Furthermore, the incorporation of interpretability analysis algorithms such as gradient-weighted class activation mapping (Grad-CAM), score-weighted class activation mapping (Score-CAM), and layer-wise relevance propagation (LRP) can reveal correlations between model attention regions and critical pathological structures, providing verifiable visual evidence for AI-driven diagnosis (25).

Although AI has demonstrated significant potential in the analysis of gastrointestinal pathology, there remains a lack of systematic research integrating “task-scale-interpretation.” Most existing studies focus solely on single-task or single-scale data training, failing to comprehensively evaluate model robustness and generalization across multi-resolution and multi-center datasets (26). Furthermore, clinical validation of model interpretability is relatively insufficient, with no established quantitative standards to assess the consistency between model-generated heatmaps and pathological decision-making (27). Achieving high accuracy while ensuring cross-scale generalization and clinical interpretability remains a pivotal scientific challenge for the practical implementation of intelligent pathology.

Building upon this rationale, we developed and validated a deep learning framework integrating multi-task learning and interpretability mechanisms to enable cross-scale automatic classification and segmentation of tumor regions and microenvironmental components in gastrointestinal cancer histopathology images. The study was conducted using two publicly available datasets, GasHisSDB (28) and GCHTID [29; dataset 10.6084/m9.figshare.25954813.v1], and systematically incorporated multi-scale preprocessing, model construction, cross-domain validation, and interpretability analysis. A unified color normalization strategy (Macenko method (30), and objective image quality control using BRISQUE (31) were applied to establish a modeling pipeline with robustness across resolutions and data sources. By introducing a Swin Transformer-based multi-task architecture (32), joint optimization of classification and segmentation was achieved. Furthermore, Grad-CAM, Score-CAM, and layer-wise relevance propagation (LRP) were incorporated to provide visually reviewable decision evidence and error-tracing cues. Blinded review by expert pathologists was additionally performed to quantify the concordance between model-highlighted regions and expert-identified diagnostic areas, thereby enhancing decision transparency and structural interpretability. The key innovation of this study lies in proposing an integrated digital pathology AI methodological framework centered on task fusion, scale generalization, and evidence visualization. This framework provides a reproducible technical pathway for model development, evaluation, and cross-source validation under multi-institutional data conditions.

## Materials and methods

### Dataset sources and sample construction

This study was conducted in accordance with the Declaration of Helsinki (2013 revision) and public data usage regulations. All data were obtained from publicly accessible databases, and no additional ethical approval was required. Two high-quality public gastric cancer histopathology datasets were used to construct classification and segmentation tasks: GasHisSDB was employed for image-level binary classification modeling and cross-resolution generalization evaluation, while GCHTID was used for multi-tissue classification and segmentation modeling. It should be noted that this study used tile-level histological images as the analytical unit. As both image-level labels and pixel-level segmentation annotations were available, a fully supervised learning paradigm was adopted for joint classification and segmentation modeling. Considering that differences may exist between public datasets and real-world clinical workflows in terms of acquisition protocols, case selection, and annotation standards, potential implications for external validity have been separately discussed in the “Limitations” section. In contrast, weakly annotated whole-slide image (WSI) classification typically relies on MIL for slide-level aggregation. Recent methodological advancements—including iterative MIL, key-instance-driven MIL, task-specific multi-level feature modeling, and hierarchical MIL—have been proposed to enhance focus on diagnostically relevant regions and improve interpretability (10–13). These methodological advances represent a distinct yet complementary research direction to our strongly annotated multi-task setting. The necessity of extending the present framework to weakly labeled WSI scenarios and incorporating MIL-based comparative validation is further discussed in the Discussion and Limitations sections. To facilitate independent reproducibility, the Methods section provides a transparent and replicable workflow. Samples were extracted directly from the original public datasets along with their corresponding annotations. Data splitting was performed using stratified random sampling, ensuring mutually independent training, validation, and test sets at a 7:1:2 ratio. Image quality control included stain normalization using the Macenko method, sharpness screening based on BRISQUE (threshold = 50), and exclusion based on tissue proportion (threshold = 40%), where tissue boundaries were automatically detected using OpenCV Canny edge detection combined with an area-threshold algorithm. In addition, a random subset of the training set was manually reviewed to confirm label consistency. A summary of inclusion and exclusion statistics is provided in **Supplementary Table S1**, and the sampling review and consistency analysis are summarized in **Supplementary Table S2**, ensuring that the experimental protocol can be directly reproduced under standardized criteria.

### Data preprocessing and quality control

All images were uniformly converted to three-channel RGB format and subjected to stain normalization using the Macenko method to reduce batch effects arising from staining variability. Subsequently, multi-dimensional quality control procedures were implemented to ensure structural integrity, discernible boundaries, and label usability. In the first stage, image sharpness was quantitatively screened using BRISQUE, and samples with BRISQUE scores > 50 were excluded as low-quality images. In the second stage, tissue proportion was calculated to remove tiles with insufficient tissue content. Tissue boundaries were automatically identified using OpenCV Canny edge detection combined with an area-threshold algorithm, and images with tissue coverage below 40% were excluded. To further mitigate the impact of potential label noise, a random subset of the training set was manually reviewed to verify annotation reliability. A summary of sample exclusion and retention statistics at each stage is provided in **Supplementary Table S1**, and the sampling review process and agreement analysis are summarized in **Supplementary Table S2**.

### Model architecture and training strategies

To accomplish classification and segmentation of gastric cancer histopathology images, we constructed a multi-level deep learning framework encompassing image-level classification, pixel-level segmentation, and joint classification–segmentation models (the overall network architecture, data flow, and loss integration are illustrated in **Supplementary Figure S1**). For the classification task, three representative architectures, ResNet-50, EfficientNet-B4, and Swin Transformer, were employed and fine-tuned using ImageNet-pretrained weights. The models output binary classification probabilities and were optimized using the binary cross-entropy (BCE) loss. The classification threshold was initially set to 0.5 and further optimized on the validation set by maximizing the F1-score. For tissue segmentation, U-Net++ and DeepLabV3+ (with ResNet-101 as the backbone) were adopted to predict nine tissue classes (TUM, LYM, STR, MUS, ADI, MUC, DEB, BACK, and NORM). A combination of class-weighted cross-entropy loss and Dice loss was used to balance pixel-level accuracy, particularly for small-volume tissue classes such as LYM and DEB. To integrate shared semantic features between classification and segmentation, a MTL model based on R2U-Net was designed. A shared encoder extracted multi-scale features, which were fed into a segmentation decoder for pixel-level prediction and, in parallel, into a global pooling and fully connected branch for image-level classification. A joint loss function was adopted during training: *L*_total_=*L*_BCE_+*L*_CE_+2·*L*_Dice_, with a weight ratio of 1:1:2. This ratio was determined in preliminary experiments based on overall validation performance and remained fixed across all experiments. Model selection was based on validation performance, and the best weights were saved to ensure consistent training and model selection criteria across methods. Residual gating mechanisms were introduced to enhance deep feature propagation, and deep supervision was applied at multiple feature scales to improve boundary delineation stability.

### Model training configuration and evaluation metrics

The original dataset was split into training, validation, and independent test sets at a ratio of 7:1:2. Model training was conducted on the training set, the validation set was used for threshold determination and optimal weight selection, and the independent test set was reserved exclusively for final performance reporting. To assess model stability, five-fold cross-validation was performed within the training-validation framework, and the mean ± standard deviation of performance metrics were reported. For the classification task, the following metrics were evaluated: Accuracy, Precision, Recall, F1-score, and area under the ROC curve (AUC). For the segmentation task, Intersection over Union (IoU) and Dice coefficient were reported. The multi-task model was evaluated using both classification and segmentation metrics, and convergence behavior and overfitting risk were further assessed using loss curves. To ensure consistency and avoid discrepancies across different implementations, all evaluation metrics were defined as follows: Precision = TP/(TP + FP), Recall = TP/(TP + FN), F1-score = 2 × Precision × Recall/(Precision + Recall), IoU = TP/(TP + FP + FN), Dice = 2TP/(2TP + FP + FN), AUC was calculated as the area under the ROC curve. These definitions were applied consistently throughout the study.

### Generalization capability and cross-scale validation

To evaluate model generalization across image scales and data sources, cross-scale and cross-dataset validation experiments were conducted. For cross-scale validation, subsets of GasHisSDB with 80-, 120-, and 160-pixel resolutions were used. Models were trained on one resolution and tested on the remaining resolutions to assess scale sensitivity and robustness. For cross-dataset validation, bidirectional cross-testing was performed between GCHTID and GasHisSDB, simulating real-world domain shifts without domain adaptation. To further assess robustness to staining variability, stain perturbation experiments were conducted under simulated H&E variations. The hematoxylin (H) channel intensity was adjusted by ±10%, and the eosin (E) channel intensity by ±15%, reflecting plausible staining fluctuations. All comparisons followed the predefined statistical testing procedures and significance criteria described in the Statistical Analysis section to ensure comparability and reproducibility across experimental settings.

### Model interpretability analysis

To elucidate the decision-making mechanisms of the model in interpreting pathological images, multiple interpretability algorithms, including Grad-CAM, Score-CAM, and LRP, were employed to generate the most discriminative activation regions within convolutional feature layers. A total of 100 model-predicted samples were selected and subjected to blind review by three pathology experts with over ten years of experience. The experts assessed the consistency between the model’s segmentation outputs and activation heatmaps in highlighting key pathological features. The expert-model agreement rate (EMAR) and Cohen’s Kappa coefficient were calculated to quantify the level of agreement. Retrospective analysis was performed on misclassified samples to compute the spatial discrepancy between model attention areas and misclassification regions, termed error-attention discrepancy (EAD), exploring whether the model exhibits excessive responses to non-pathological regions. To validate the robustness of model explanations, Grad-CAM heatmaps before and after staining perturbations were overlapped and analyzed for structural consistency and visual stability.

### Statistical analysis methods

This study focuses on algorithmic performance evaluation using publicly available histopathological tile images. Statistical analyses were primarily conducted to quantify differences in model performance, stability, and interpretability consistency, rather than to support clinical population inference. For metrics obtained from cross-validation or repeated experiments, normality was first assessed. Parametric tests (e.g., independent-samples t-test or paired t-test) were applied when normality assumptions were satisfied; otherwise, nonparametric tests (e.g., Kruskal-Wallis test) were used. For multi-model comparisons, one-way ANOVA followed by Bonferroni *post hoc* correction was performed when assumptions were met. Correlation analyses were conducted using Pearson’s correlation coefficient. Agreement was evaluated using Cohen’s kappa with corresponding 95% confidence intervals (CIs). Confidence intervals for primary performance metrics were estimated using bootstrap resampling. All statistical tests were two-sided, with a significance level set at α = 0.05. In both the main text and **Supplementary Materials**, mean values, standard deviations, and percentage-point differences (Δ) were reported to reflect effect sizes, thereby avoiding overreliance on *P*-values alone. To ensure reproducibility under standardized criteria, key thresholds and procedures were explicitly predefined in the Methods section, including stain normalization, BRISQUE threshold (50), tissue proportion threshold (40%), stratified data splitting (7:1:2), five-fold cross-validation, cross-scale and cross-dataset validation settings, and H/E channel perturbation ranges. These specifications are aligned with **Supplementary Figure S1**; **Supplementary Tables S1, S2** to ensure that replication does not depend on implicit implementation assumptions.

## Results

### Data quality control significantly enhances image clarity and tissue structure consistency

To maintain conciseness in the main text, auxiliary analyses, including sample screening statistics, label consistency review, and comprehensive performance comparisons, are provided in the **Supplementary Materials**. **Supplementary Table S1** summarizes image quality control and sample retention statistics; **Supplementary Table S2** presents the results of random sampling review and agreement analysis; and **Supplementary Table S3** reports performance comparisons between the proposed method and baseline models. The main text focuses on key findings and their clinical implications. To ensure consistency in image quality and tissue structure representation for model training, a two-stage quality control procedure was applied to the two public GC pathology datasets (GasHisSDB and GCHTID). First, image sharpness was quantitatively screened using BRISQUE (threshold = 50). Second, tissue proportion assessment was performed to exclude tiles with insufficient tissue coverage. Detailed exclusion and retention statistics for each stage are provided in **Supplementary Table S1**.

To further remove samples with inadequate tissue representation, tissue boundaries were automatically identified using OpenCV Canny edge detection combined with an area-threshold algorithm, and tiles with tissue coverage below 40% were excluded. This process eliminated 1,950 invalid images located primarily at the peripheral regions of WSIs or containing predominantly background. After two rounds of quality control, 84,220 high-quality H&E images with clear tissue structures were retained in the GasHisSDB dataset, and 15,050 high-quality images were retained in the GCHTID dataset, covering eight distinct tissue categories (e.g., TUM, STR, LYM, ADI) (**Supplementary Table S1**). Overall, approximately 6.3% of images were excluded. The optimized dataset exhibited markedly improved image clarity and structural consistency, thereby reducing the impact of low-quality samples and label noise and providing a reliable foundation for subsequent model training, validation, and generalization experiments.

**Figure 1:**
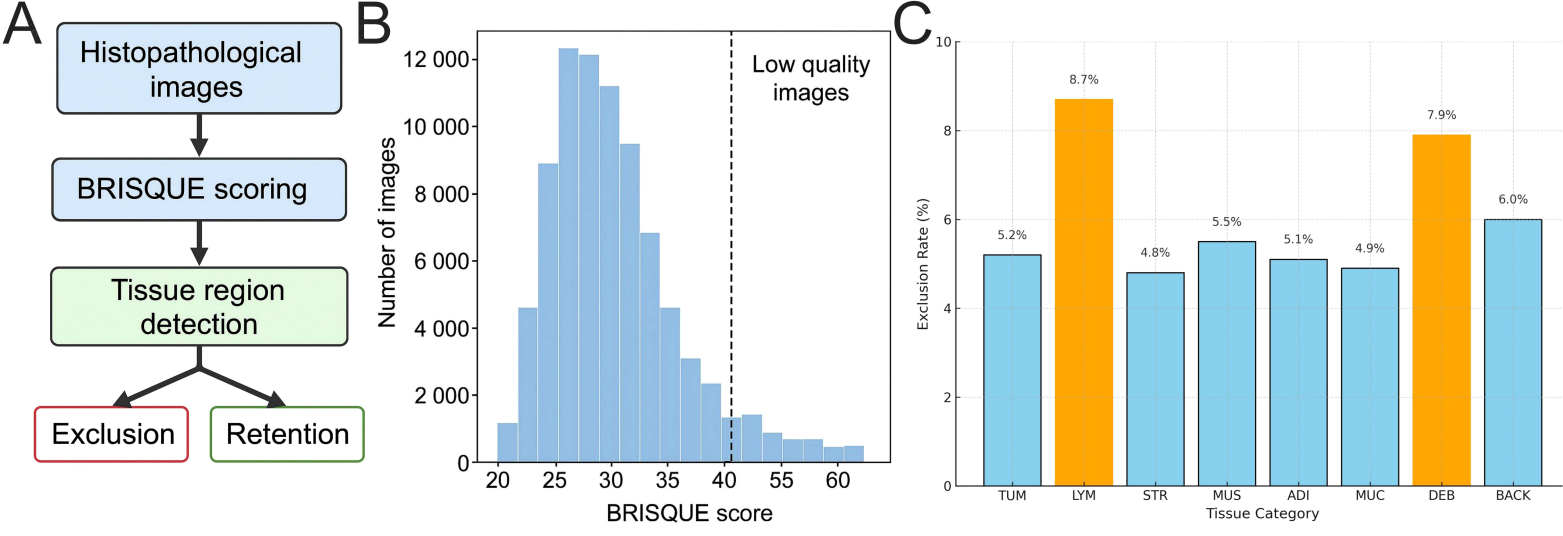
Workflow of pathological image quality control and sample screening results. **(A)** Schematic representation of the overall workflow for dataset preprocessing and quality control, comprising two stages: BRISQUE clarity assessment and tissue proportion detection. **(B)** Histogram distribution of BRISQUE scores in the GasHisSDB dataset, predominantly within the 30–45 range, with scores above 50 considered low-quality samples. **(C)** Distribution of image exclusion ratios across tissue types in the GCHTID dataset, with marginally higher contamination rates observed for LYM and DEB categories.

Overall, this phase of the quality control strategy effectively reduced data noise caused by imaging artifacts, staining biases, and uneven tissue proportions. It ensured that the input data for DL models adhered to a uniform standard in terms of spatial resolution and tissue structure, thus enhancing the trainability and generalization performance of the models (**Figure 1**).

### Enhancing annotation accuracy and consistency of tissue labels through expert review

**Figure 2:**
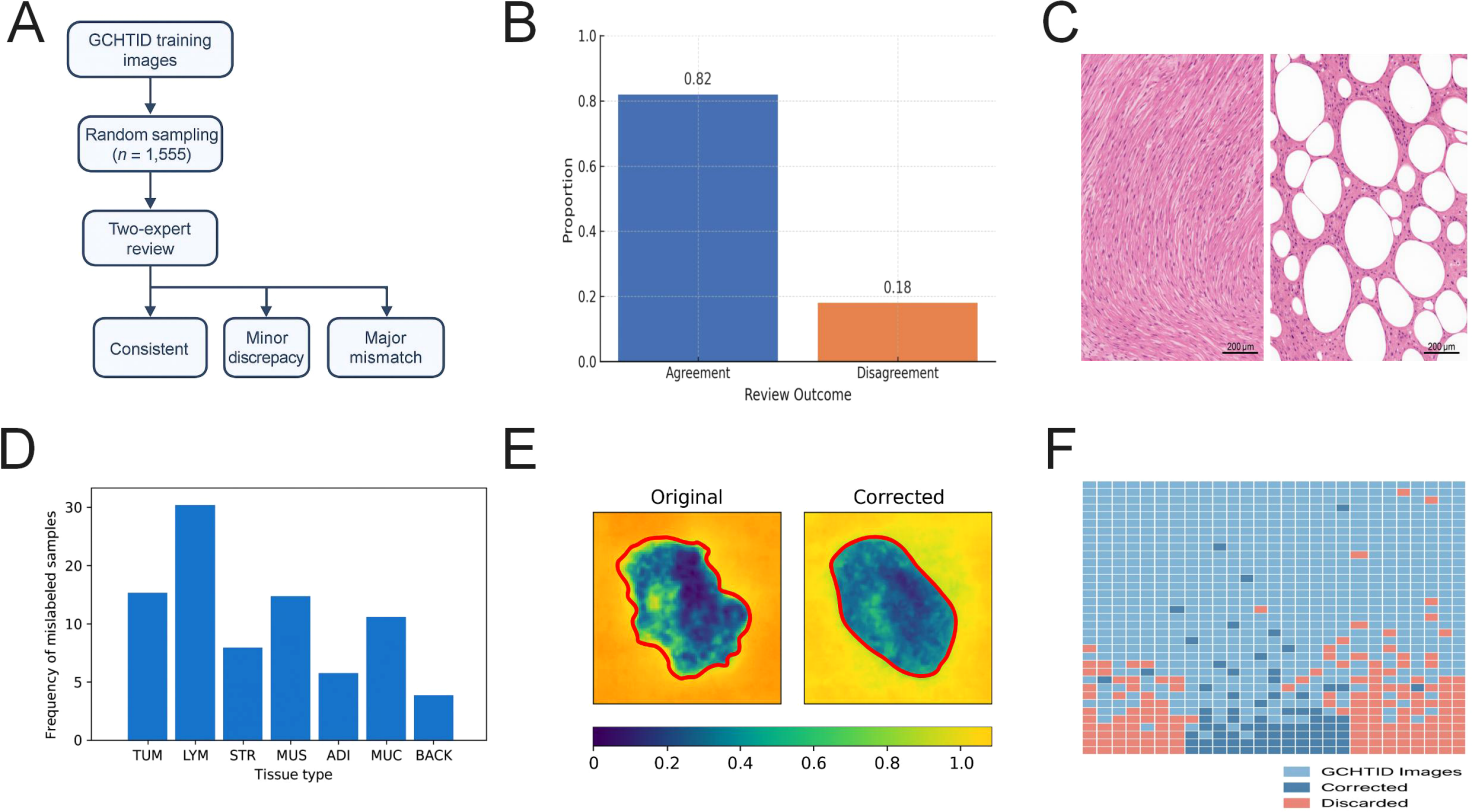
Label consistency review improves annotation accuracy and inter-expert agreement. **(A)** Workflow diagram of label consistency review: 1,555 images randomly sampled from the GCHTID training set were reviewed by two experts; review results were categorized into three types: consistent, partially deviating, and severely mismatched. **(B)** Evaluation of label consistency using Cohen’s Kappa coefficient, Cohen’s Kappa = 0.82, indicating good agreement between expert judgments and original labels. **(C)** Illustration of typical label inconsistency samples; the left image has an original label of STR, while experts consistently judged it as MUC, showing a clear structural discrepancy. **(D)** Bar chart depicting the distribution frequency of mislabeled samples across eight tissue types; MUC, STR, and LYM categories exhibit relatively higher deviation rates. **(E)** The heatmap visualization comparison before and after label correction highlights discrepancies between annotated region boundaries and actual tissue structures. **(F)** The spatial distribution and proportion of corrected and excluded samples within the overall GCHTID dataset indicate that post-correction label deviation rates significantly decreased.

To ensure the accuracy and professional consistency of tissue annotations in the GCHTID dataset, a random subset of the training set was selected for blinded review by two independent pathologists (**Figure 2A**). Agreement metrics, sample size, and adjudication outcomes are summarized in **Supplementary Table S2** (**Figure 2B**), demonstrating overall good consistency.

For samples identified as having evident labeling errors or expert disagreement, predefined rules were applied to either correct the labels or exclude the samples, thereby reducing the impact of label noise on model training. The corresponding numbers and processing proportions are detailed in **Supplementary Table S2**.

Mislabeling was primarily observed in tissue categories with relatively ambiguous morphological boundaries or frequent transitional regions. The distribution of affected categories and representative examples are shown in **Figures 2C-E**, with statistical summaries provided in **Supplementary Table S2**.

### Stratified sampling ensures highly balanced distribution of organizational categories

**Figure 3:**
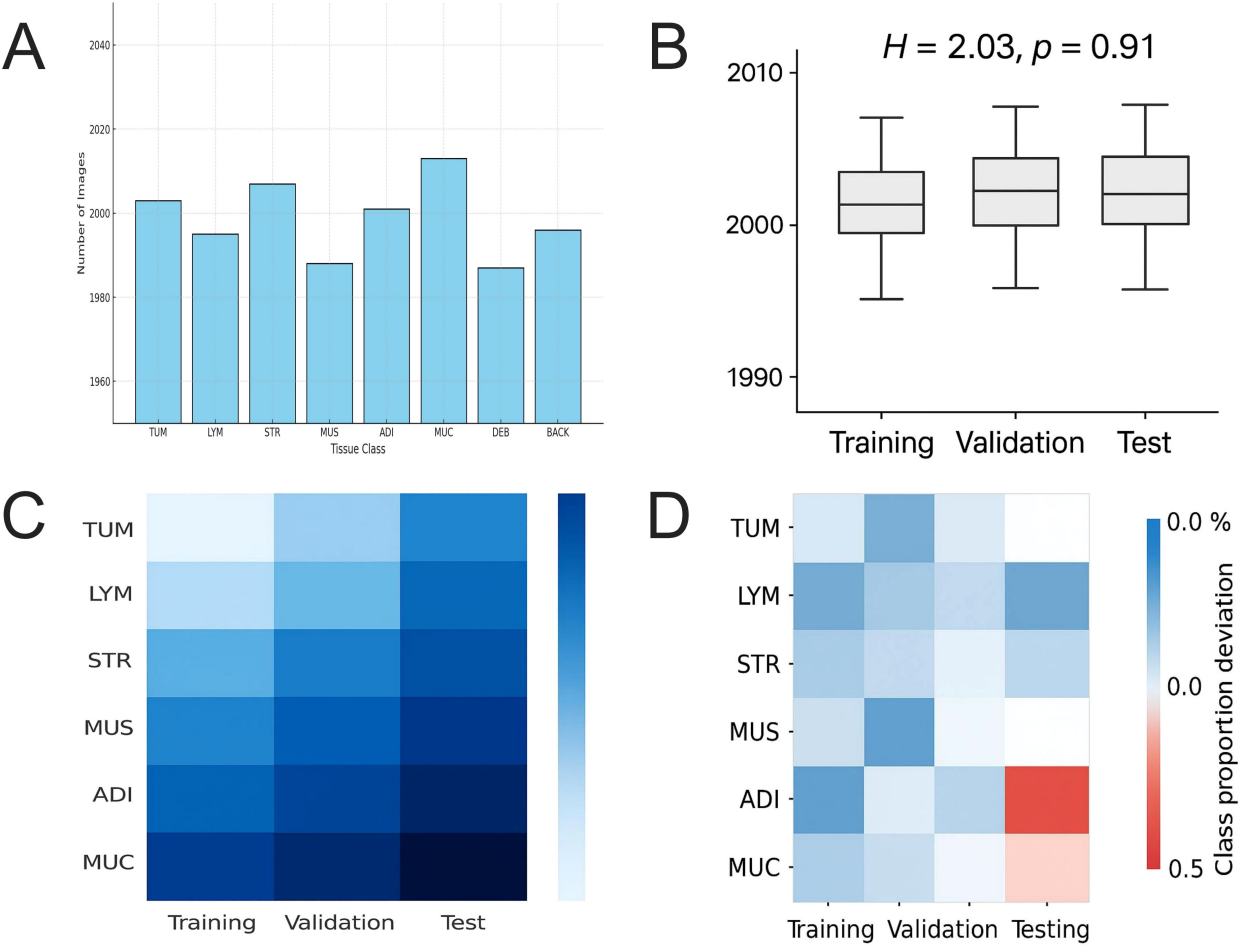
Analysis of the balance in tissue category distribution within the GCHTID training dataset. **(A)** A bar chart illustrating the distribution of sample counts for eight tissue categories in the training dataset, with each category containing approximately 2,000 ± 15 samples, indicating near-ideal balance. **(B)** The Kruskal-Wallis test results, which compare sample counts across categories in the training, validation, and test datasets, show no significant differences (H = 2.03, *p =* 0.91). **(C)** Heatmaps represent tissue category distributions across three datasets. Darker colors indicate higher sample counts, demonstrating good symmetry in the overall distribution. **(D)** Bias matrix for sample count proportions of tissue categories across training, validation, and test datasets; most categories exhibit a division bias below ± 0.5%, with the largest bias observed in the DEB category (+0.7%).

To maintain consistent representation of tissue categories during model training, a stratified random sampling strategy was adopted to construct the training, validation, and test sets. This approach ensured approximately balanced distributions of the eight tissue classes across all subsets. The range of sample sizes and variation metrics for each category are summarized in **Supplementary Table S2** (**Figure 3A**).

Nonparametric tests were further conducted to assess distribution consistency among the three subsets. No statistically significant differences were observed, supporting the statistical balance of the dataset partitioning (**Supplementary Table S2**, **Figure 3B**).

Heatmaps and proportion deviation matrices additionally confirmed symmetric class distributions and controlled bias across subsets (**Figures 3C, D**). Detailed numerical results and deviation limits are provided in **Supplementary Table S2**.

### Swin transformer achieves superior performance in gastric tissue classification

To evaluate the discriminative capabilities of various mainstream network architectures in the task of gastric tissue image classification, a systematic comparison was conducted on the GasHisSDB subset with a resolution of 160×160 pixels. Three image classification models—ResNet-50, EfficientNet-B4, and Swin-T—were examined. All models were initialized with pre-trained parameters from ImageNet and fine-tuned under the same training dataset and hyperparameter settings. The test set comprised 17,000 quality-controlled images.

**Figure 4:**
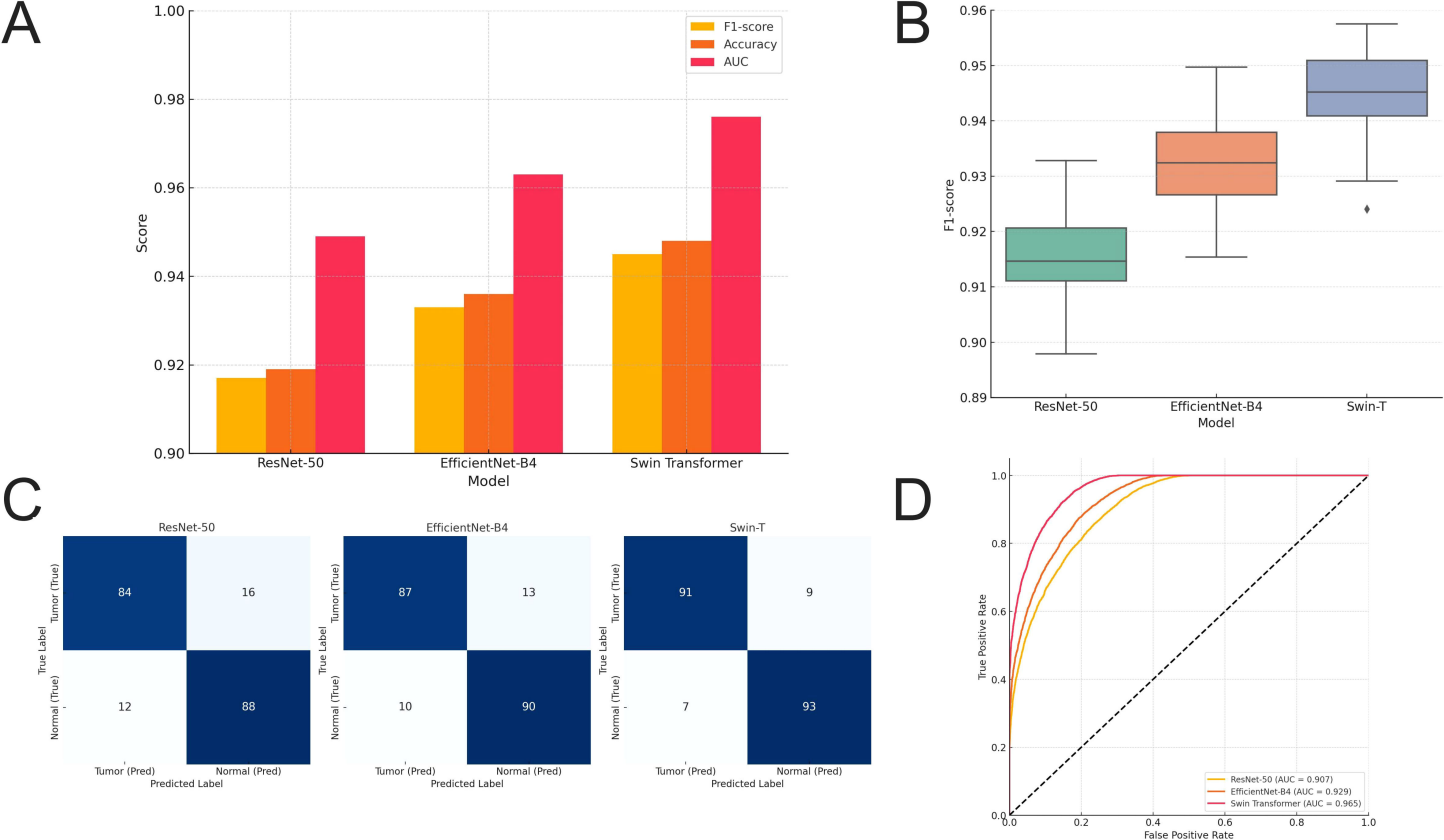
Performance comparison of three image classification models on gastric cancer tissue images. **(A)** The average performance of the three models in terms of F1-score, Accuracy, and AUC, with Swin-T significantly outperforming the other two CNN models. **(B)** Results of one-way ANOVA (F = 12.47, *p <* 0.001), showing that Swin-T has a statistically significant advantage in F1-score compared to ResNet-50 and EfficientNet-B4 (Bonferroni-corrected *p <* 0.01). **(C)** Comparison of confusion matrices among the three models, where Swin-T exhibits the lowest misclassification rate for the Tumor class and a significant reduction in misclassification of non-tumor tissues. **(D)** Comparison of ROC curves for each model, with Swin-T demonstrating higher discriminative sensitivity in high-threshold regions (upper right quadrant), achieving the highest AUC of 0.965.

In terms of overall performance, Swin Transformer achieved the highest F1-score (0.945 ± 0.008), outperforming EfficientNet-B4 (0.933 ± 0.009) and ResNet-50 (0.917 ± 0.010) by 1.2 and 2.8 percentage points, respectively (**Figure 4A**). The corresponding Accuracy values were 0.948, 0.936, and 0.919, while the AUC values were 0.965, 0.929, and 0.907, respectively. One-way ANOVA indicated a statistically significant difference in F1-score distributions among the three models (F = 12.47, *p* < 0.001). Bonferroni-adjusted *post hoc* comparisons confirmed that Swin-T significantly outperformed both CNN-based architectures (both *p <* 0.01, **Figure 4B**). It should be noted that these improvements represent percentage-level gains. Their potential clinical relevance should be interpreted in the context of misclassification patterns, the proportion of morphologically ambiguous samples, and the practical workload in real-world diagnostic workflows. A summary of the primary classification performance metrics is provided in **Supplementary Table S3**.

From the perspective of classification decisions, Swin-T achieved a Precision of 0.957 and a Recall of 0.934 in the Tumor category, indicating its stronger discriminative ability in more structurally complex tumor regions. The model’s confusion matrix (**Figure 4C**) shows that Swin-T exhibited the lowest error rate in normal tissue classification, especially demonstrating enhanced robustness in lightly stained regions, thereby reducing misclassification.

To explore the discriminative ability of model output distributions, receiver operating characteristic (ROC) curves for the three models were plotted (**Figure 4D**). Results indicated that Swin-T exhibited superior cumulative discriminative ability in the high-CI (0.8-1.0), with the curve closer to the top left corner, suggesting higher tolerance for ambiguous boundaries or heterogeneous regions. Overall, the findings demonstrate that Swin-T, based on its Transformer architecture, delivers more stable recognition performance and cross-sample generalization ability under conditions of morphological complexity, texture heterogeneity, and staining diversity in pathological images.

### DeepLabV3+ achieves higher overall segmentation performance and improved structural consistency in multi-tissue segmentation

In the GCHTID tissue image test set, the performance of U-Net++ and DeepLabV3+ (with ResNet101 as the backbone) was compared as the core architectures for pixel-level segmentation tasks, focusing on their ability to accurately identify multi-class tissue regions and reconstruct structural details. Both models were trained for 100 epochs using standard data partitioning, with weighted cross-entropy and Dice loss for joint optimization, and evaluated under identical input dimensions (224×224) and image augmentation strategies.

**Figure 5:**
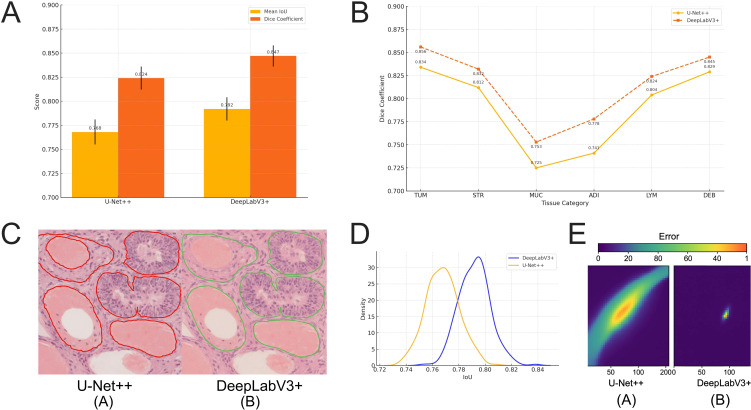
Comparison of overall performance between U-Net++ and DeepLabV3+ models in gastric tissue image segmentation. **(A)** Overall comparison of the two models in terms of mean IoU and Dice metrics, with DeepLabV3+ significantly outperforming U-Net++ (*p <* 0.05). **(B)** Dice metric comparison across different tissue categories, where DeepLabV3+ exhibits superior performance in all categories, particularly in MUC and ADI classes. **(C)** Examples of segmentation results, showing boundary fractures in U-Net++ results on the left and more continuous edge reconstruction by DeepLabV3+ on the right. **(D)** Kernel density estimation curves for IoU distribution on the test set, indicating a more concentrated and stable performance by DeepLabV3+. **(E)** Heatmap of typical misclassification regions, demonstrating larger error areas at heterogeneous tissue boundaries for U-Net++, while DeepLabV3+ exhibits more compact and focused responses.

DeepLabV3+ demonstrated superior performance in modeling overall tissue structures, achieving an average IoU of 0.792 ± 0.012 and a Dice coefficient of 0.847 ± 0.011 on the test set. In comparison, U-Net++ achieved an average IoU of 0.768 ± 0.013 and a Dice coefficient of 0.824 ± 0.012 (**Figure 5A**). The performance difference between the two models was statistically significant according to an independent samples t-test (*p <* 0.05), indicating that DeepLabV3+, with its incorporation of atrous convolution and feature pyramid structures, possesses stronger spatial representation and multi-scale modeling capabilities in complex backgrounds. The primary segmentation performance metrics of the relevant models are summarized in **Supplementary Table S3**.

From the perspective of single-class tissue evaluation, DeepLabV3+ achieved IoU values of 0.812 and 0.785 for large-volume tissues such as TUM and STR, respectively, and also performed well in irregular boundary structures like the MUC class (IoU = 0.753). **Figure 5B** presents a bar chart comparing the Dice coefficients across different classes, showing that DeepLabV3+ achieved performance improvements in all categories, particularly in mucous and adipose tissue recognition, with improvements exceeding 3.6%. Furthermore, representative image segmentation results (**Figure 5C**) reveal that U-Net++ often exhibited fractures or blurry boundaries in edge regions, whereas DeepLabV3+ provided more continuous and clearly defined structural reconstructions.

**Figure 5D** displays the IoU distribution density plots of both models across the entire test set, where DeepLabV3+ exhibits a higher and more concentrated peak with a distinct right skew, indicating superior overall stability. **Figure 5E** further visualizes the pixel mask distribution of misclassified regions, showing that U-Net++ presents noticeably higher errors at the overlapping tissue boundaries (the interface between LYM and DEB), suggesting that it still suffers from boundary ambiguity when handling tissue heterogeneity.

Taken together, DeepLabV3+, owing to its semantic awareness and feature context fusion mechanism, achieves more accurate structural reconstruction in tissue pixel segmentation tasks and demonstrates greater generalization capability and robustness in multi-class scenarios.

### DeepLabV3+ maintains higher segmentation accuracy in small-volume and boundary-blurred tissues

**Figure 6:**
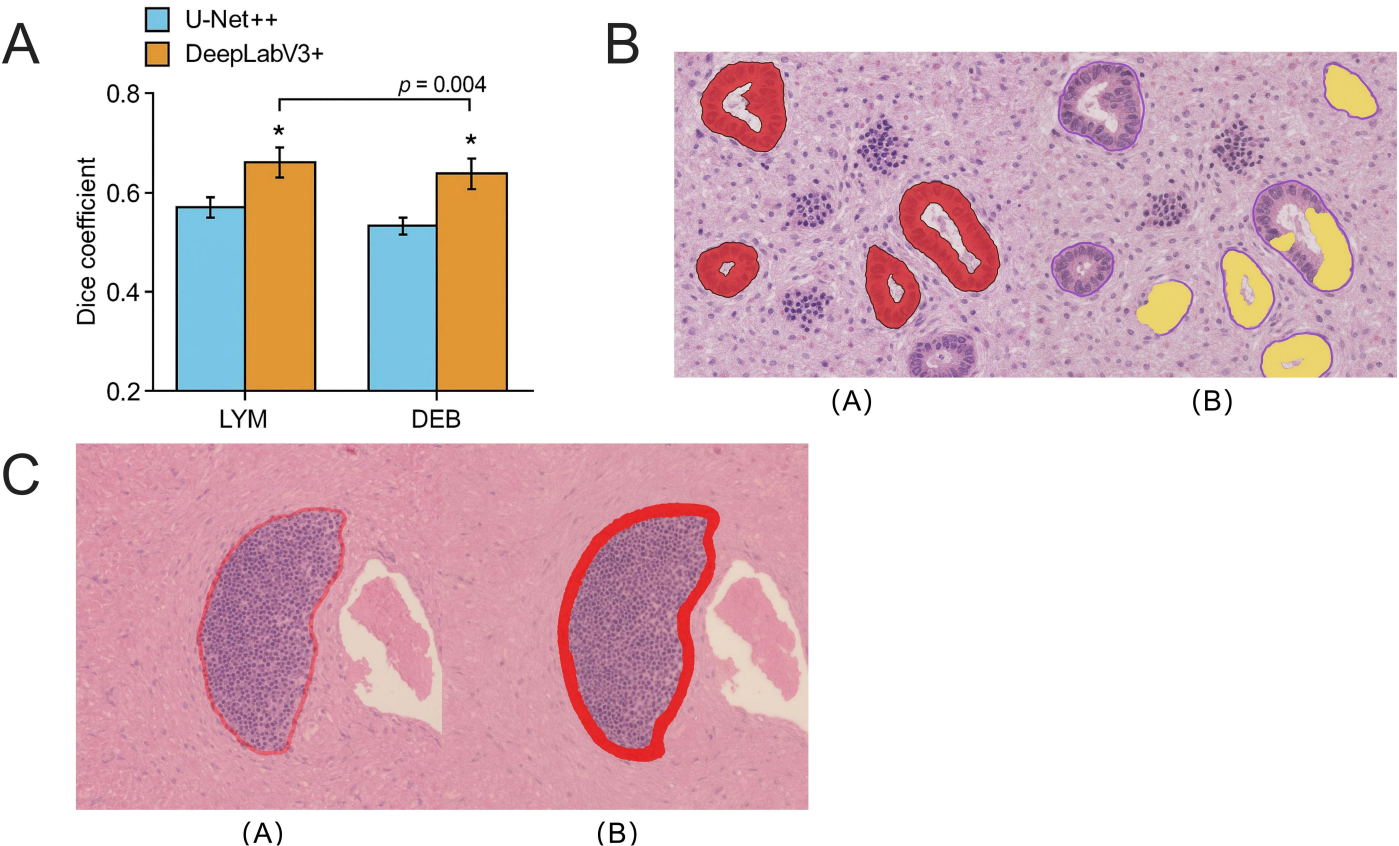
The segmentation performance advantage of DeepLabV3+ in small-volume tissue categories. **(A)** Model comparison on the Dice metric for small-volume tissue categories LYM and DEB, where DeepLabV3+ significantly outperformed U-Net++ (LYM: *p =* 0.004; DEB: *p =* 0.016). **(B)** The segmentation result comparison for typical small-volume tissue images shows that U-Net++ exhibited boundary shifts and structural fractures in high-background images, whereas DeepLabV3+ predicted regions were more concentrated and adhered better to true boundaries. **(C)** Error response analysis: The misjudged regions (in red) at the edges were significantly fewer for DeepLabV3+ compared to U-Net++, with an average boundary deviation reduction of 2.8 pixels, indicating higher boundary matching accuracy. The asterisk symbols indicate statistical significance levels: p < 0.05.

Although LYM and DEB occupy an extremely low proportion in tissue images, exhibit blurred boundaries, and demonstrate high morphological variability, differences between the two models in the segmentation of small-volume tissues are still observable. On the GCHTID test set, DeepLabV3+ achieved a Dice coefficient of 0.781 ± 0.021 for the LYM category, significantly outperforming U-Net++ (0.747 ± 0.025). This difference was statistically significant as validated by an independent sample t-test (*p =* 0.004), indicating that the atrous convolution structure offers notable advantages in modeling small-scale regions (**Figure 6A**).

Additionally, DEB tissues, characterized by discontinuous morphology and incomplete boundaries, exhibited greater variability in performance with the U-Net++ model (Dice = 0.703 ± 0.028). In contrast, DeepLabV3+ achieved a Dice coefficient of 0.739 ± 0.023 for the same category, reflecting an improvement of over 3.6%. **Figure 6B** illustrates the segmentation results of both models on typical small-volume tissue images, showing that U-Net++ predictions exhibited edge misclassification and tissue fragmentation, especially in high-background-noise images where false positives were more likely. DeepLabV3+, however, preserved more intact tissue morphology in the same regions and exhibited a more restrained response to artifacts.

To further quantitatively evaluate the models’ sensitivity to small-volume tissue boundaries, prediction-error heatmaps and edge response maps were constructed, and the spatial overlap ratio between them was calculated. Results showed that DeepLabV3+ reduced the erroneous response rate in LYM boundary regions by approximately 15.2% and decreased the average boundary deviation distance (BDD) by 2.8 pixels (**Figure 6C**), demonstrating its ability to better capture boundary contour features under complex backgrounds, thereby enhancing the consistency of small-structure recognition.

These findings suggest that, compared to conventional architectures, the atrous receptive field design and pyramid context fusion mechanism of DeepLabV3+ enable more robust segmentation performance in small-proportion, low-contrast tissue regions in images, making it an important structural choice for addressing real tissue heterogeneity and scale variability.

### MTL enhances joint performance and synergy between classification and segmentation

To further integrate image-level and pixel-level contextual information, this study developed a multi-task DL framework based on the R2U-Net backbone, optimizing both image classification and tissue component segmentation tasks. The model features a shared encoder core with separate decoder branches for image-level classification and pixel-level segmentation. The joint loss function is defined as a combination of BCE, Cross-Entropy, and Dice Loss, with weight ratios determined through preliminary experiments to be 1:1:2.

**Figure 7:**
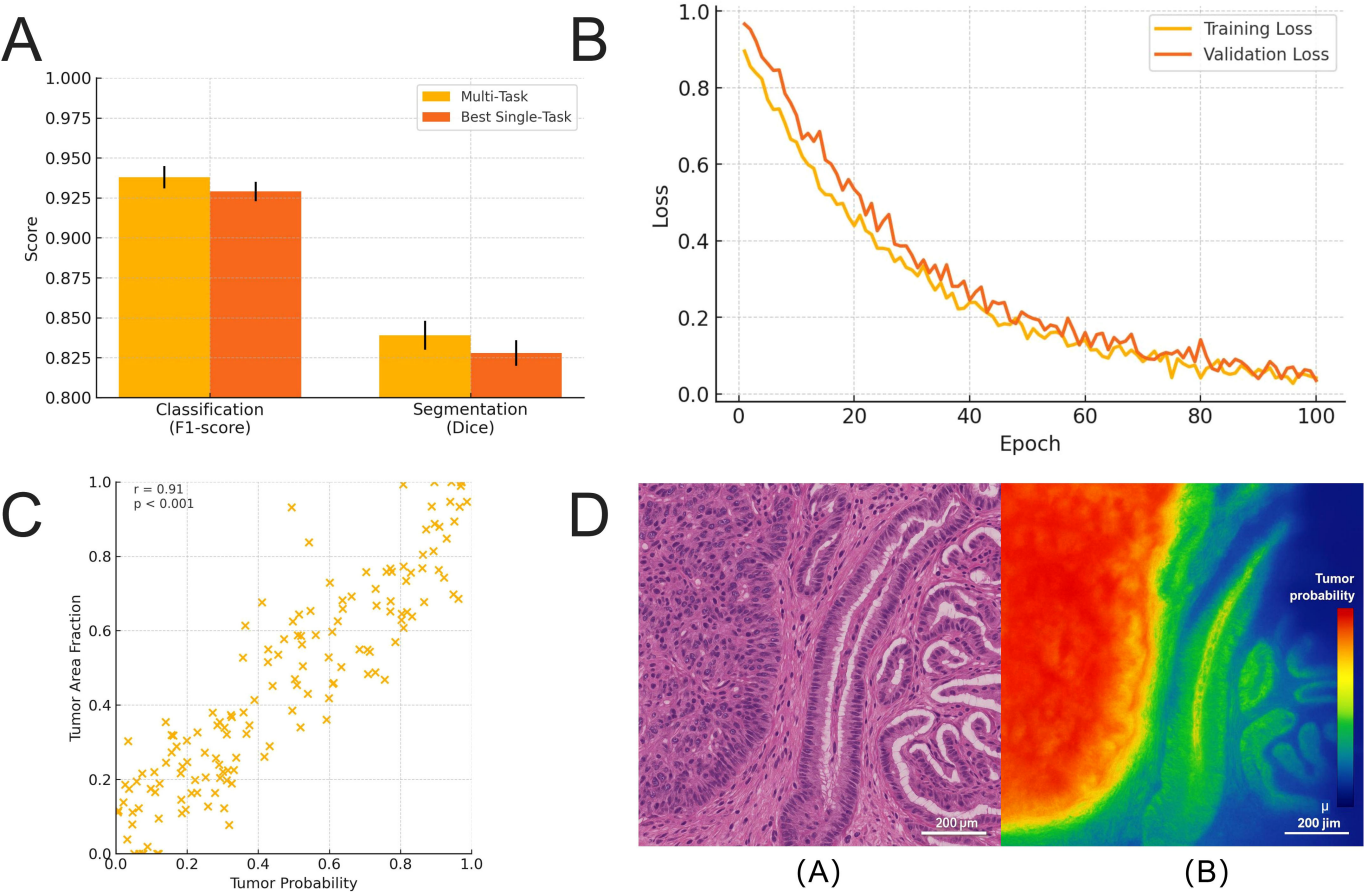
Comprehensive performance improvement of the MTL model in gastric tissue image recognition. **(A)** Performance comparison of the MTL model in image classification (F1-score) and pixel-level segmentation (Dice) tasks, showing improvements over the best single-task models (*p <* 0.05). **(B)** Joint loss reduction curves during training and validation phases, where the MTL model demonstrates better stability and convergence in later stages. **(C)** Scatter plot depicting the Pearson correlation between image-level tumor prediction probabilities and pixel-level tumor area, with a correlation coefficient of r = 0.82 (*p <* 0.001), indicating semantic coupling between tasks. **(D)** Visualization examples of MTL outputs, presenting classification heatmaps and segmentation masks, which exhibit enhanced boundary recognition and structural consistency at tissue junctions.

On the GCHTID test set, the multi-task model achieved an F1-score of 0.938 ± 0.007 for image-level classification, while the pixel-level tissue segmentation task reached a mean Dice coefficient of 0.839 ± 0.009 (**Figure 7A**). Compared with single-task models, the multi-task framework demonstrated performance metrics within the same order of magnitude for both tasks, while exhibiting greater stability in output consistency and structural alignment. Independent-samples t-tests indicated statistically significant differences between the multi-task and single-task models in key performance metrics for both tasks (*p <* 0.05). It is important to note that these differences represent percentage-level improvements. Rather than constituting a qualitative leap, they are more appropriately interpreted as robustness gains in transitional regions and weak-boundary scenarios. Their potential clinical value lies in reducing boundary-related misclassification risk, improving the efficiency of reviewing challenging cases, and providing more structurally coherent support for interpretable visualizations.

Training process analysis revealed that the loss function of the multi-task model converged more stably, particularly in the mid-to-late training stages (epoch 50~100), where the joint loss exhibited a steady decline and minimal fluctuations in validation loss (**Figure 7B**). This suggests that joint optimization contributes to mitigating overfitting and underfitting during parameter updates. Furthermore, in terms of model output consistency, high-confidence regions in the segmentation results were highly consistent with classification predictions, and the image-level Tumor probability showed a significant positive correlation with the pixel-level TUM area (r = 0.82, *p <* 0.001, **Figure 7C**), indicating effective synergy between the two tasks at the structural representation level.

Qualitative performance analysis, as illustrated in **Figure 7D**, shows the integrated outputs of the multi-task model for typical tissue images. Compared to single-task models, the multi-task architecture demonstrated superior boundary awareness and classification sensitivity in heterogeneous regions, such as the TUM-MUC interface. Predictions were more compact, noise responses were reduced, and lesion coverage completeness was improved. Overall, the joint model maintained the ability to discern overall image structures while enhancing fine-grained perception of local tissue boundaries and microstructures, showcasing the discriminative advantages of cross-scale integration.

### Model robustness across multi-resolution conditions with boundary detection slightly affected by scale reduction

To systematically evaluate the stability and generalization ability of classification models under varying image resolution conditions, an experimental framework was constructed based on the Swin-T to assess scale adaptability. Performance was independently tested on three sub-datasets of GasHisSDB (80×80, 120×120, 160×160). By maintaining a constant training set resolution (160×160) without any fine-tuning or compensation, the model’s prediction robustness under varying input resolutions was examined.

**Figure 8:**
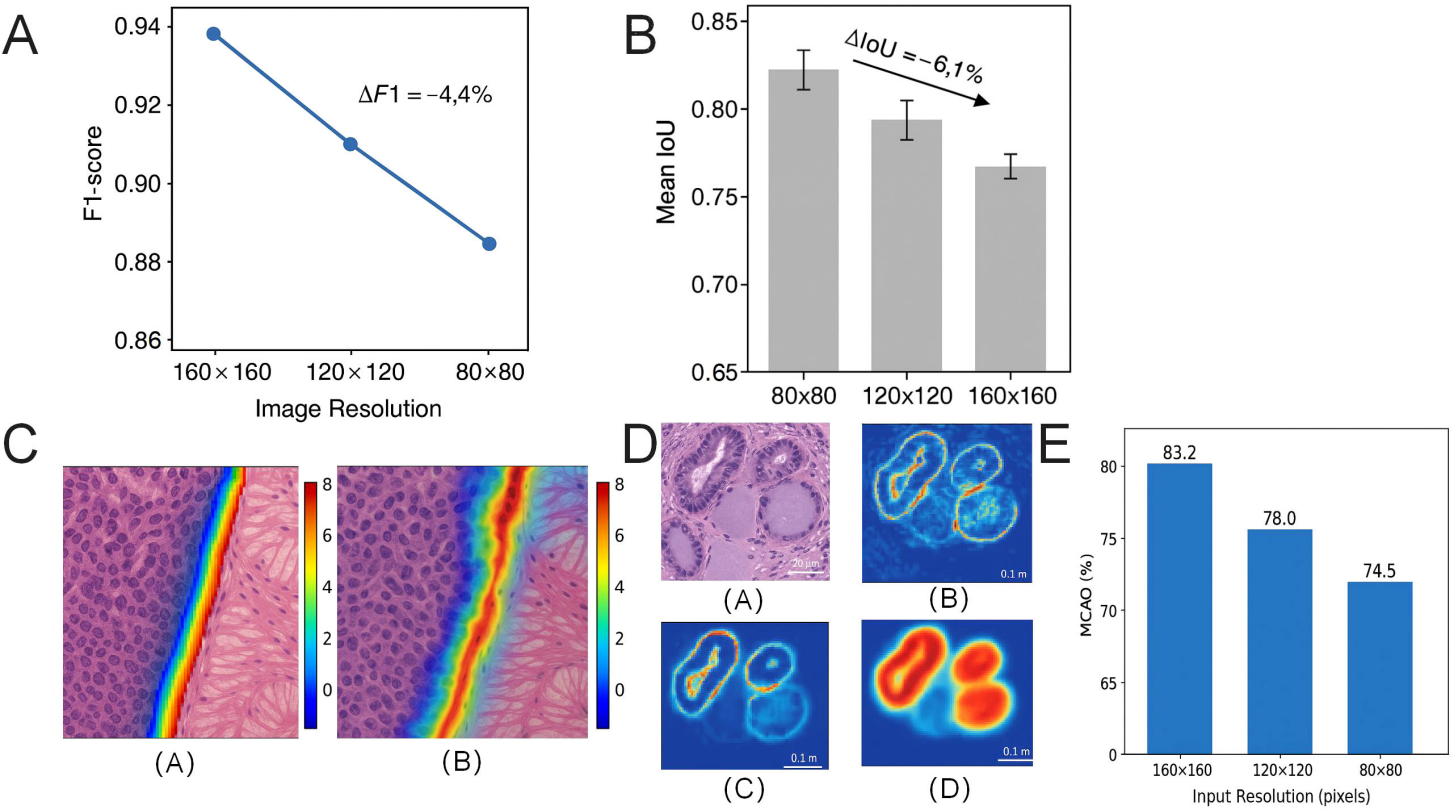
Classification performance and boundary recognition stability of the Swin-T model under varying image resolutions. **(A)** Variation in F1-score for the Swin-T model under three input resolutions: 160×160, 120×120, and 80×80. Performance decreases progressively with lower resolution, with ΔF1 reaching -4.4%. **(B)** Comparison of mean IoU across different resolutions, showing the most significant decline in boundary region IoU, with ΔIoU = -6.1%. **(C)** Example heatmap of boundary segmentation errors; misclassification regions become concentrated at edges as resolution decreases, exhibiting a “smoothing” tendency. **(D)** Comparative heatmaps of misclassification probability under varying resolutions; at 80×80 resolution, the model’s recognition ability for mucinous structures significantly deteriorates. **(E)** Comparison of the spatial overlap between class activation maps and actual tumor regions shows that reduced resolution markedly decreases the model’s perception accuracy.

Under high-resolution conditions (160×160), the Swin-T model achieved an F1-score of 0.945 ± 0.008. When the image resolution was reduced to 120×120, the F1-score decreased to 0.928 ± 0.010, and further decreased to 0.901 ± 0.013 at 80×80 resolution. The overall performance drop was ΔF1 = -4.4% (**Figure 8A**), showing a gradual decline with decreasing input scale. The IoU metric exhibited a more pronounced decrease, from 0.851 at 160×160 to 0.790 at 80×80, with ΔIoU = -6.1%, particularly amplifying errors in tissue boundary regions (**Figure 8B**).

Further spatial visualization analysis of segmentation errors in tumor tissue (TUM) boundary regions revealed a “smoothing” trend in model-predicted boundaries under low-resolution input, leading to the loss of fine details in true boundary structures (**Figure 8C**). The pixel error area in blurred boundary regions significantly increased, with the average offset distance rising from 4.2 pixels to 6.7 pixels (*p <* 0.01). As shown in **Figure 8D**, low-resolution input also affected the semantic recognition ability of tumor tissue local structures in classification tasks, with increased misclassification rates concentrated in regions where texture details were missing, such as mucinous degeneration and degradation zones.

Additionally, the CAM of the model at three resolutions was computed, and the overlap degree of their response to tumor regions (mean class activation overlap, MCAO) was evaluated. From 160×160 to 80×80, the average overlap between CAM and the ground truth mask decreased from 83.2% to 74.5%, suggesting that the model’s perceptual regions gradually deviated from critical pathological areas (**Figure 8E**).

In summary, although the Swin Transformer exhibits a certain degree of cross-scale structural adaptability, its performance remains constrained by the spatial resolution of the input images. Notably, degradation is observed in boundary awareness and fine-grained texture modeling capabilities at lower resolutions. This finding suggests that reduced input resolution diminishes the model’s sensitivity to structural boundaries and subtle textural details.

### Swin-T demonstrates strong cross-institutional transfer and domain generalization capability

To evaluate the adaptability of deep classification models across different data sources, a bidirectional cross-validation experimental framework was designed, utilizing GCHTID and GasHisSDB as two sets of heterogeneous data. These datasets exhibit significant differences in sample source institutions, staining processes, image textures, and tissue distributions. The objective was to investigate the cross-domain transfer performance of the Swin-T under conditions without retraining.

**Figure 9:**
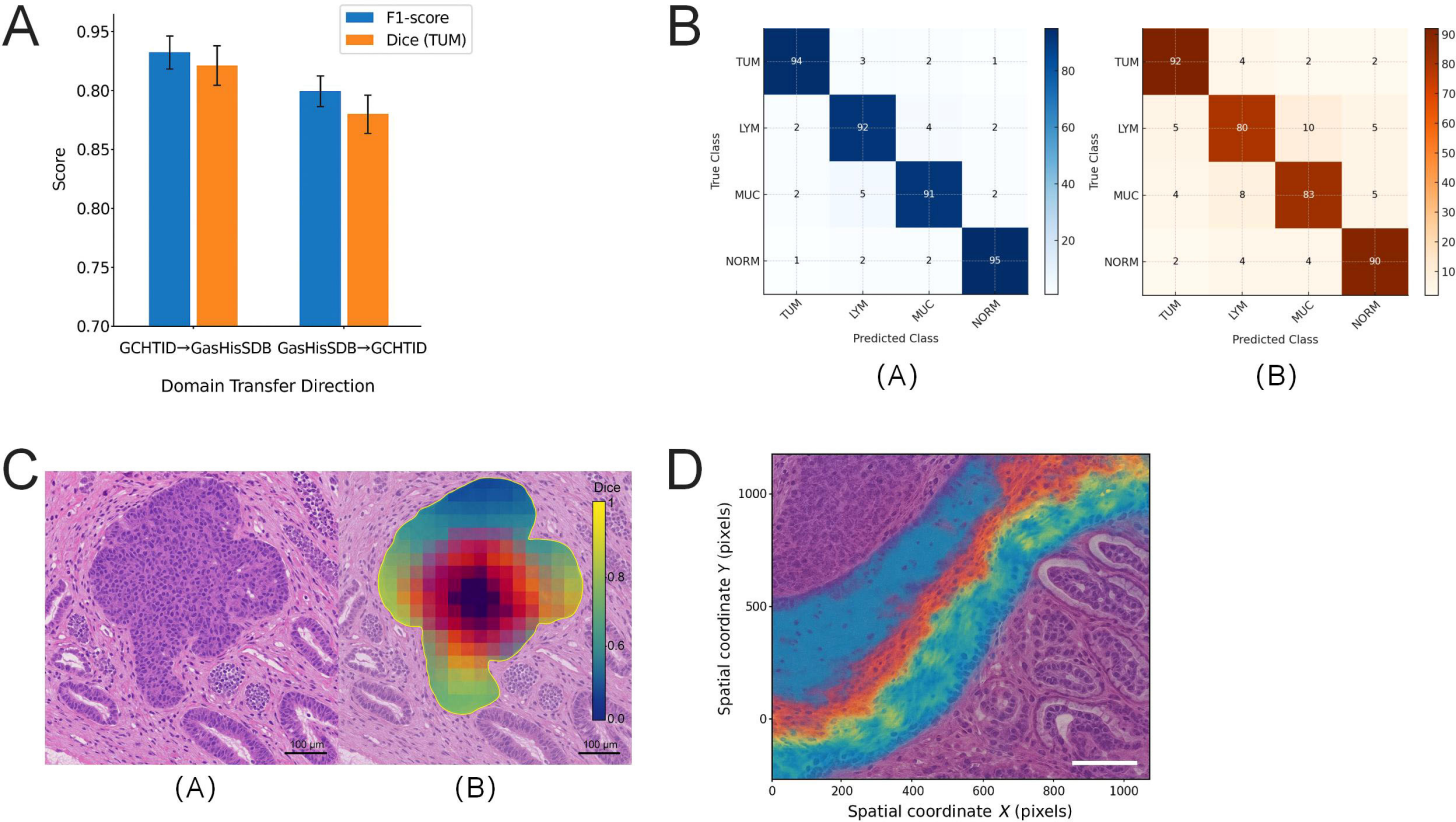
Performance of the Swin-T model in bidirectional cross-domain transfer on different gastric cancer tissue data sources. **(A)** Comparison of model F1-score and Dice for TUM class under the bidirectional cross-domain transfer framework, showing better test performance in the GCHTID→GasHisSDB direction compared to the reverse path (ΔF1 = 2.6%; ΔDice = 3.2%). **(B)** The confusion matrix heatmap comparison illustrates domain-specific tissue class misclassification shifts, with increased misclassification rates for LYM in the reverse path. **(C)** Dice visualization map of TUM response areas in typical test images, demonstrating clearer boundaries and more focused responses under GCHTID training conditions. **(D)** The cross-domain Dice error heatmap reveals a concentrated spatial distribution of misclassification in background regions with lighter staining and structural junctions. This pattern reflects the impact of differences in staining styles and structural representation between data sources on transfer performance.

In the experiments, forward transfer testing was first conducted, using GCHTID as the training set and GasHisSDB as the test set. Under this setup, the model achieved an F1-score of 0.902 ± 0.015 on GasHisSDB, with a Dice coefficient of 0.831 ± 0.013 for TUM tissue. In reverse testing, using GasHisSDB as the training set and GCHTID as the test set, the model achieved an overall F1-score of 0.876 ± 0.018 and a Dice coefficient of 0.799 ± 0.017 for TUM tissue (**Figure 9A**). The performance difference between the two tests indicates mild domain shift (ΔF1 ≈ 2.6%, ΔDice ≈ 3.2%), yet the model maintained acceptable generalization capability overall.

**Figure 9B** presents the confusion matrix visualization for both rounds of experiments. Under the GCHTID training condition, the model demonstrated better control over misclassification rates for non-tumor tissues. Conversely, when trained on GasHisSDB, the model exhibited a noticeable decline in differentiation capability for LYM and MUC classes, indicating that differences in training data coverage of tissue heterogeneity can influence the model’s generalization behavior.

To further understand the structural sources of performance shift, response regions for the TUM class in different test sets were extracted, and Dice spatial response maps were calculated (**Figure 9C**). It was observed that, when trained on GCHTID and tested on GasHisSDB, the model was better at capturing complete tumor contour regions. However, in the reverse direction, boundary response areas showed some shrinkage, particularly in MUC transition zones and low-staining regions, resulting in missed detections.

**Figure 9D** displays the inter-domain Dice difference heatmaps of the two groups, visualizing the response consistency of identical labels on a per-image basis. The heatmaps indicate that errors are primarily concentrated in regions with faint staining, irregular backgrounds, and invasive tumor margins. Such tissues exhibit notable deviations in imaging style across different data sources, forming the main cause of cross-domain performance degradation.

Despite the systematic variations in staining patterns, tissue contours, and image sources, Swin-T maintains high classification and segmentation performance without domain adaptation, demonstrating strong cross-institutional transfer potential and suitability for multi-scenario pathological assistance systems.

### Robust prediction and boundary consistency of transformer architecture under H&E staining variations

H&E staining variations pose a significant challenge to cross-batch generalization in DP images. To evaluate model robustness under staining variability, we developed an H&E perturbation testing framework that simulates channel perturbations on the GCHTID test dataset images. Specifically, the H channel intensity was adjusted by ± 10%, and the E channel intensity was perturbed by ± 15%, simulating color variations within the range of real-world staining fluctuations.

**Figure 10:**
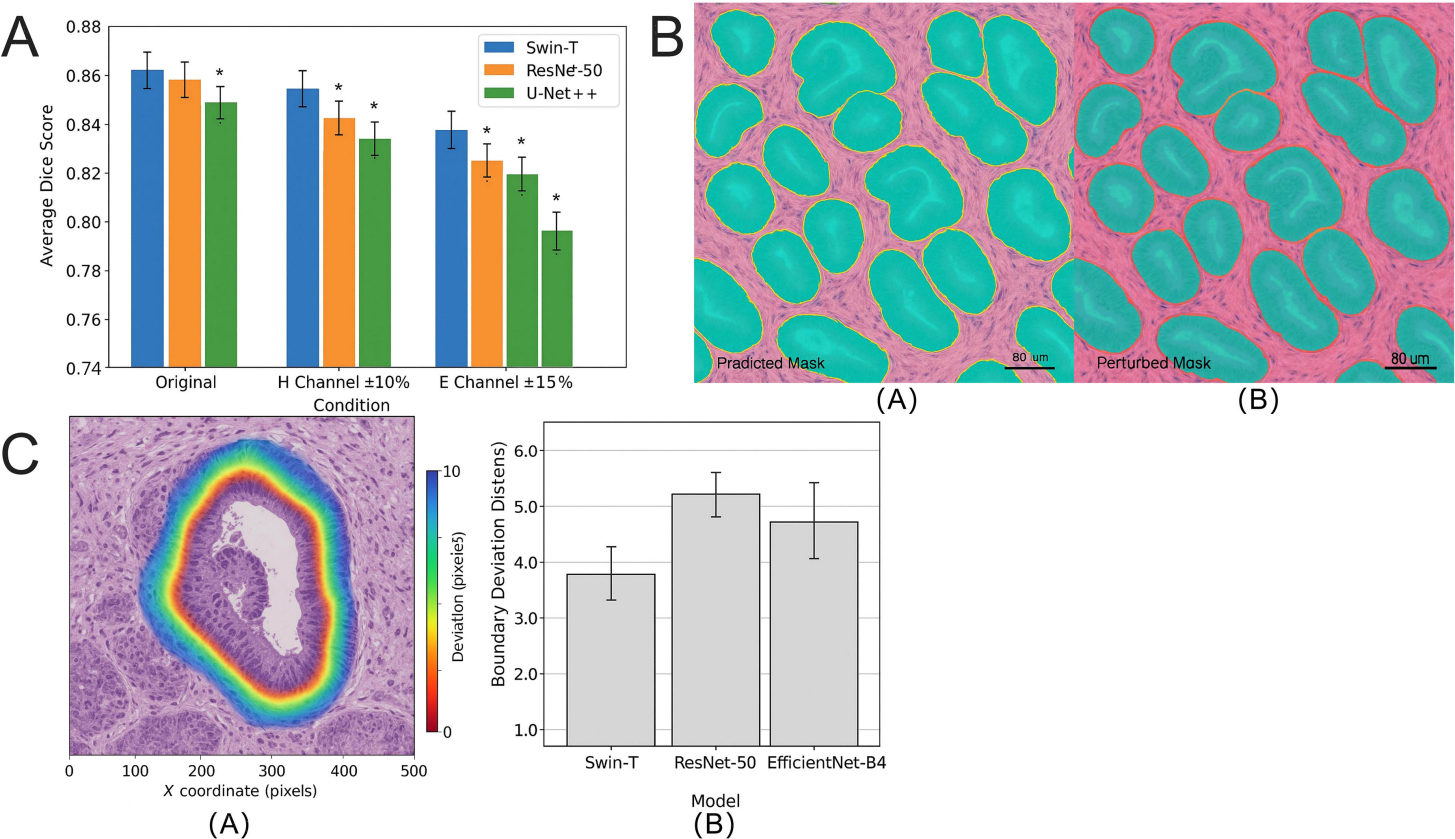
Robustness evaluation of Swin-T under H&E staining perturbations. **(A)** Changes in average Dice scores of the model under original, H-channel perturbations (± 10%), and E-channel perturbations (± 15%) conditions. The performance degradation of Swin-T is significantly less than that of ResNet-50 and U-Net++ (*p <* 0.01). **(B)** Segmentation comparison images of typical cases, analyzing the boundary stability of predicted masks before and after perturbations. Swin-T exhibits negligible boundary shrinkage in predicted regions. **(C)** Heatmaps of spatial displacement and BDD distributions for predictions before and after perturbations. The mean boundary displacement of Swin-T is significantly smaller than that of CNN-based architectures, demonstrating superior structural awareness robustness. The asterisk symbols indicate statistical significance levels: p < 0.05.

Without fine-tuning the model, the Swin-T achieved an average Dice score of 0.847 ± 0.011 on original images. Under H channel perturbations, the average Dice score decreased to 0.823 ± 0.014, and under E channel perturbations, it further declined to 0.812 ± 0.016 (**Figure 10A**). The performance under both perturbations was significantly lower than that on original images (*p <* 0.01, paired t-test), yet remained within a high accuracy range, demonstrating strong staining robustness.

Compared to traditional CNN architectures (ResNet-50 and EfficientNet-B4), Swin-T exhibited smaller performance degradation under staining perturbations (ResNet-50: ΔDice = -5.8%; Swin-T: ΔDice = -2.4%), indicating superior generalization adaptability to color space variations. **Figure 10B** further illustrates segmentation differences in typical images before and after perturbations. U-Net++ showed notable shrinkage in boundary response regions and expansion of error areas under mild perturbations, whereas Swin-T maintained clear boundaries and consistent segmentation contours, with predicted regions closer to the real tissue structures.

To quantify changes in boundary sensitivity, we calculated the average BDD and pixel-level difference maps before and after perturbations (**Figure 10C**). Swin-T exhibited an average boundary deviation of only 3.1 pixels under E channel perturbations, significantly lower than ResNet-50 (5.4 pixels, *p =* 0.006), and maintained stable structural responses across multiple tissue junction regions.

In summary, Transformer architectures demonstrate high stability under color perturbations and maintain robust classification and segmentation performance in unstable staining environments, making them more suitable for deployment in multi-center and cross-platform clinical pathology DP analysis scenarios.

### The model achieves accurate identification in most tissue types but shows structural confusion between TUM and MUC

In the GCHTID test dataset, the image-level multi-class classification model based on the Swin-T backbone demonstrated consistent performance, achieving high accuracy in identifying various tissue types. To further analyze its classification characteristics and misclassification tendencies across different tissue structures, a multi-class confusion matrix was constructed, and precision metrics were utilized for detailed analysis.

**Figure 11:**
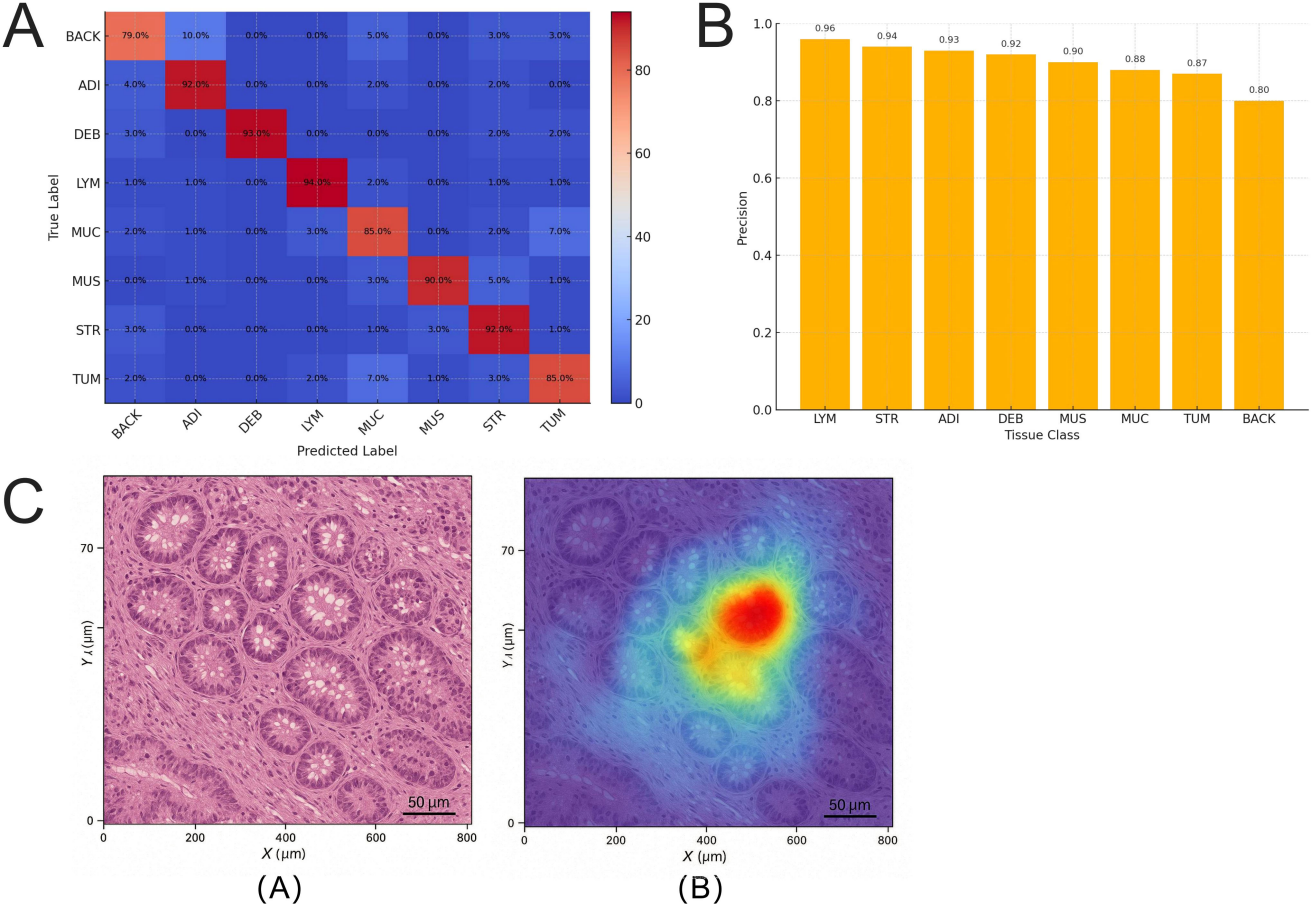
Confusion matrix and misclassification analysis for multiclass tissue image classification. **(A)** The confusion matrix of eight tissue classes output by the Swin-T on the GCHTID test set. The confusion rate between TUM and MUC reaches 7.3%, with LYM being the most accurately classified class. **(B)** The bar chart of Precision metrics for each class shows that LYM, STR, and ADI achieve classification precision above 0.92, while BACK has the highest misclassification rate, often erroneously categorized as tissue types with similar structural densities. **(C)** Spatial heatmaps of activation maps for misclassified samples, where TUM is misclassified as MUC, are concentrated in regions with blurred boundaries and mucin infiltration, indicating model perception bias in continuous tissue representation.

The model achieved the highest precision in the LYM category, with a precision of 0.958 ± 0.006 and a recall of 0.944, accompanied by the lowest misclassification rate (**Figure 11A**). LYM exhibits distinct morphological features such as dense nuclei and clear boundaries, enabling the model to respond with high consistency. Conversely, BACK showed a higher misclassification rate, with a recall of only 0.791, primarily due to the similarity in staining between certain background areas and low-density adipose tissue or motion artifacts, leading to confusion with ADI or MUC categories (**Figure 11B**).

The confusion rate between TUM and MUC reached 7.3%, representing a significant bottleneck in current classification performance. These two categories exhibit structural transitions or overlaps in certain pathological subtypes, particularly in secretory adenocarcinoma regions, where tumor cells are surrounded by abundant mucin-like structures. This results in unclear boundaries during classification, leading to judgment bias. **Figure 11C** illustrates the spatial characteristics of Class Activation Map overlaps in typical misclassification cases, revealing that samples of TUM misclassified as MUC often have activation regions located in the central or transitional zones of the tissue, lacking clear boundary support. This indicates that the current model requires optimization in handling tissue heterogeneity and microscopic structural continuity.

Additionally, the confusion matrix shows that the model performs well in identifying tissues with clear structures and distinct morphologies, such as STR (stroma), ADI (adipose), and DEB (debris). The precision for these three categories exceeds 0.92, demonstrating the model’s strong capability in distinguishing tissues with well-defined morphological boundaries.

Overall, the Swin-T classification model exhibits robust performance across most tissue types, particularly excelling in identifying small-volume structures. However, challenges remain in handling tissue continuity and weak boundary expression. Future improvements may involve integrating boundary-awareness mechanisms or category-specific feature enhancement modules to further enhance cross-tissue classification accuracy.

### Swin-T combined with score-CAM significantly enhances model structural focus and interpretive consistency

To systematically evaluate the spatial attention capabilities and structural consistency of models in identifying image discriminative regions, two mainstream visual explanation methods—Grad-CAM and Score-CAM—were employed to visualize the prediction results of Swin-T and ResNet-50 on the GCHTID test set, with pixel-level comparison to the ground truth tissue region labels.

**Figure 12:**
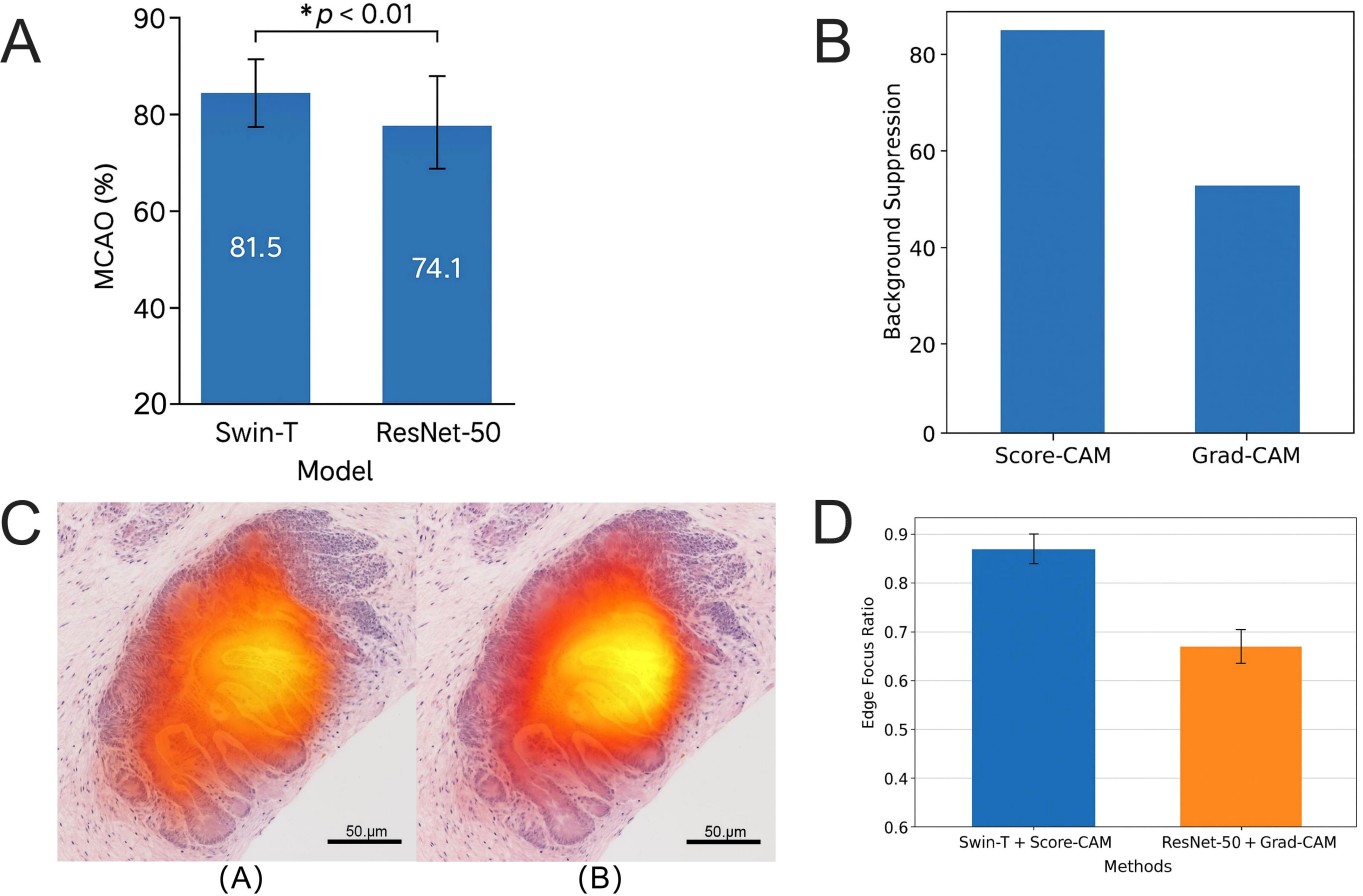
Analysis of spatial consistency and structural focus of model discriminative regions under different visualization methods. **(A)** Comparison of the MCAO based on Grad-CAM, where Swin-T achieves an average MCAO of 81.5%, significantly outperforming ResNet-50’s 74.1% (*p <* 0.01). **(B)** Comparison of heatmap focus performance between Score-CAM and Grad-CAM on the Swin-T model, showing that Score-CAM improves background interference suppression and edge activation precision. **(C)** Activation heatmaps for the same TUM image using different model+visualization method combinations, where Swin-T + Score-CAM demonstrates more compact attention regions with boundaries closely aligning to actual tissue contours. **(D)** Distribution of EFR and centroid offsets for different methods, with the Swin-T + Score-CAM combination outperforming the ResNet-50 + Grad-CAM combination in structural consistency metrics. The asterisk symbols indicate statistical significance levels: p < 0.05.

Grad-CAM analysis revealed that the mean spatial overlap of activation regions (MCAO) between the regions generated by the Swin-T model and the pathological annotation regions was 81.5% ± 4.2%, significantly outperforming ResNet-50’s 74.1% ± 5.1% (**Figure 12A**). The difference between the two models was statistically significant, as confirmed by a paired t-test (*p <* 0.01), suggesting Swin-T exhibits stronger structural recognition consistency in tissue-level feature focus and lesion region response.

Compared to Grad-CAM, Score-CAM further enhances the spatial focus of activation regions by utilizing a gradient-free scoring mechanism to strengthen the expression of high-response regions. In the Swin-T model, the regional concentration of Score-CAM heatmaps improved by approximately 11.2%, and the area of false activations decreased by over 15% (**Figure 12B**). This method demonstrated superior noise suppression capabilities in low-contrast images, particularly exhibiting stable edge structure expression for highly heterogeneous tissues such as TUM and LYM.

**Figure 12C** illustrates the overlay effects of saliency heatmaps for the same pathological image under different models and visualization methods. The Swin-T + Score-CAM combination generated high-response regions at the lesion edges in the central area of the image, accurately covering the tumor annotation regions. In contrast, ResNet-50 + Grad-CAM heatmaps exhibited an outward expansion trend, with some attention regions extending into normal tissue structures, posing a risk of excessive response.

To further quantify the local interpretive capabilities of different methods, the edge focus ratio (EFR) and centroid shift distance were calculated for each heatmap. It was found that the average EFR for Swin-T + Score-CAM in TUM-class images was 0.87, significantly higher than ResNet-50 + Grad-CAM’s 0.72. The average centroid shift distance was only 3.2 pixels (**Figure 12D**). These results indicate that Swin-T is more structurally accountable for its discriminative decisions and exhibits higher positional accuracy in pathological regions.

In summary, the combination of Transformer structures with Score-CAM achieves higher precision in the interpretation of discriminative regions. The attention regions generated not only exhibit stronger focus but also demonstrate higher spatial consistency with actual tissue structures, providing robust support for the clinical interpretability of models.

### The focus areas of model interpretability highly align with pathological experts’ assessments

To further validate the clinical interpretability of the model’s prediction results, we designed a blind evaluation experiment to assess expert-model agreement. Three clinical experts, each with over 10 years of independent pathology diagnostic experience, independently and blindly reviewed 100 model-predicted images and their associated visualized heatmaps (Grad-CAM + segmentation masks). They determined whether the model’s focus areas corresponded to the critical structures identified during their clinical interpretations. Each expert provided a binary judgment (‘consistent’ or ‘inconsistent’) for each image and documented any specific pathological factors contributing to potential discrepancies.

**Figure 13:**
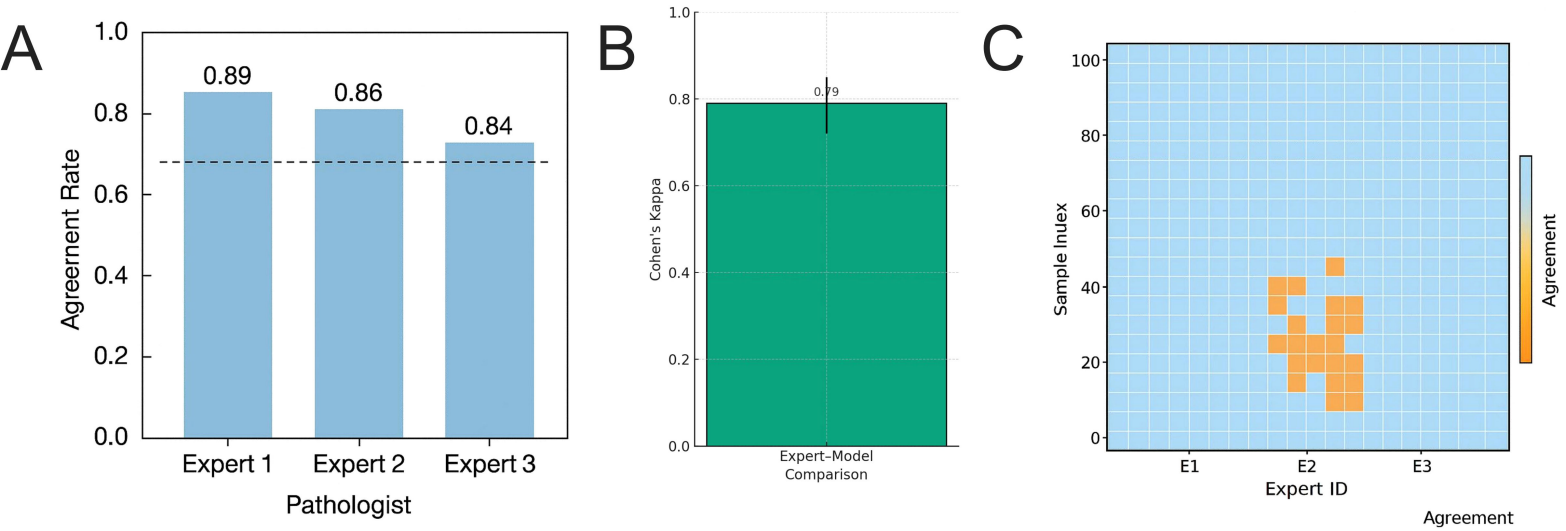
Expert-model interpretability, consistency, blind evaluation results, and differential sample analysis. **(A)** Distribution of consistency judgments by three pathology experts for 100 model-generated images, with an average EMAR value of 0.864, indicating a high overall agreement trend. **(B)** Cohen’s Kappa consistency coefficient analysis, showing substantial agreement between the three experts and the model, with a Kappa value of 0.79. **(C)** Heatmap of consistent and inconsistent labels among experts, revealing that 12% of images exhibit pairwise judgment discrepancies, primarily concentrated in regions with blurred boundaries.

The final statistical analysis revealed that the average EMAR across the three experts was 0.864 ± 0.060 (**Figure 13A**). The highest agreement among experts was 0.89, and the lowest was 0.84, demonstrating good stability of the evaluation results among experts. Cohen’s Kappa coefficient was employed to further assess the agreement level between the experts and the model, yielding a value of 0.79 (95% CI: 0.72-0.85), indicating substantial agreement (**Figure 13B**).

**Figure 13C** displays the distribution of label consistency among three experts for the same model outputs, where the majority of samples were evaluated consistently across all three experts, while a small portion (approximately 12%) showed pairwise disagreement. Further analysis revealed that the discrepancies were primarily concentrated in the following types of images: (1) those with infiltrative tumor margins and indistinct tissue boundaries; (2) mucinous adenocarcinoma samples in which TUM and MUC regions overlapped substantially; and (3) atypical adipose areas (ADI) with staining similar to the background (BACK), which were prone to misclassification. These results indicate that the attention regions generated by the Swin-T model under its explainability mechanism were highly consistent with the pathologists’ perception of diagnostically critical structures, supporting its use as visual evidence for case review and error tracing in research settings. However, response shifts persisted in regions with complex boundary structures and high heterogeneity, suggesting that the model’s attention mechanism requires further optimization through integration of expert knowledge.

### Misclassification samples primarily caused by structural boundary blurring and staining variations leading to model attention shift

To deeply understand the causes of attention mechanism shifts underlying model classification or segmentation errors, we propose the error-related attention displacement (EAD) metric. This metric quantifies the Euclidean distance between the predicted attention center and the true annotation center within visualized heatmaps, and performs comparative analysis against correctly predicted samples.

**Figure 14:**
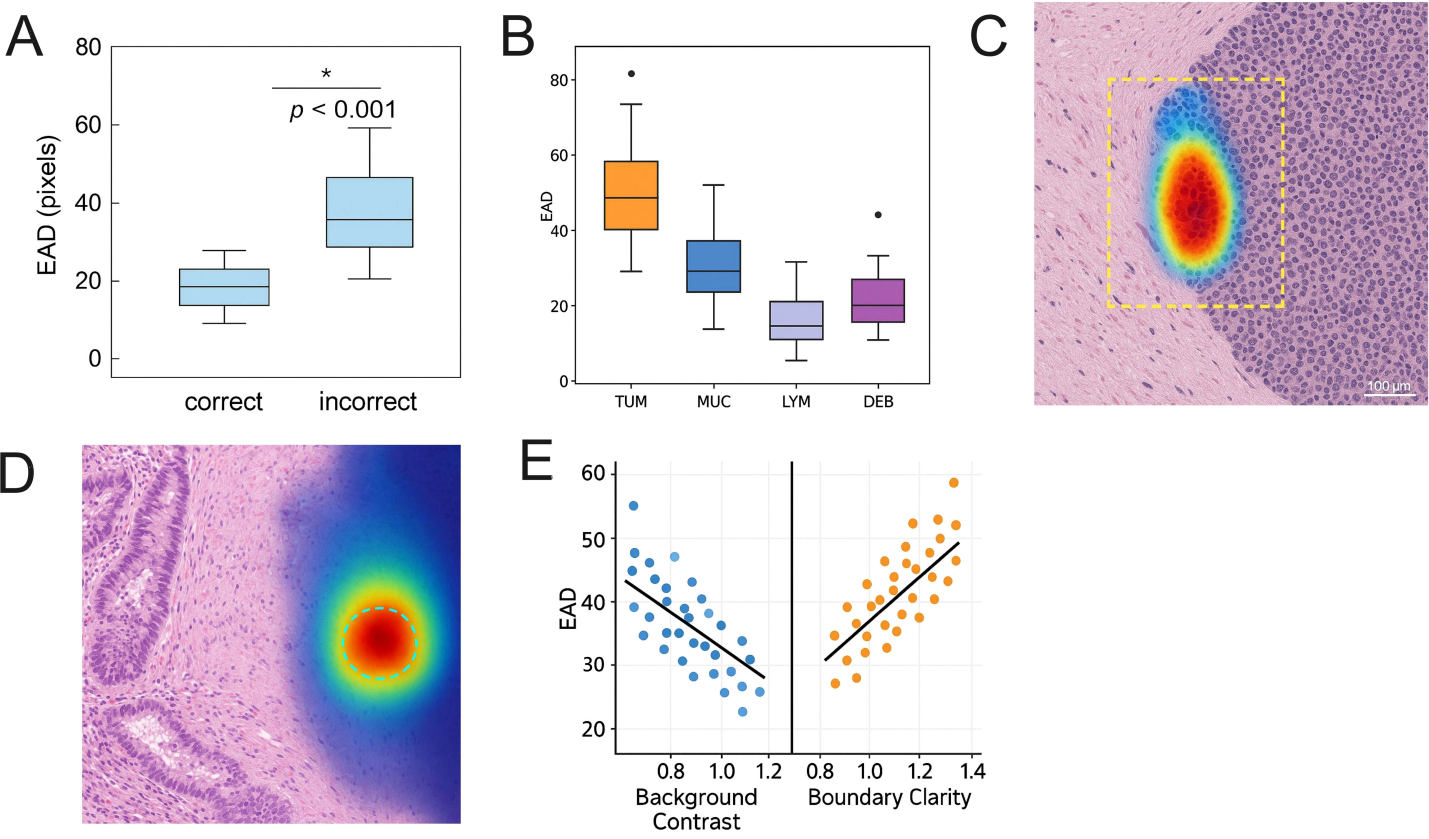
Analysis of model misclassification, attention region deviation, and structural response mechanism interpretation. **(A)** Comparison of EAD distribution between correctly and incorrectly predicted samples, with the mean EAD for misclassified samples reaching 42.6 pixels, significantly higher than that for correctly predicted samples (*p <* 0.001). **(B)** Boxplot of EAD distribution across different tissue categories, showing misclassifications concentrated at TUM/MUC boundaries, ambiguous LYM structures, and peripheral fragmented regions. **(C)** Example of tumor boundary misclassification, where activation regions deviate significantly from the actual tumor region. **(D)** The activation shift induced by staining perturbation causes the model response regions to focus on non-lesion areas. **(E)** Regression analysis of EAD with image quality control factors (contrast and boundary sharpness), indicating that the model’s attention mechanism tends to deviate from core tissue areas under scenarios of reduced image quality. The asterisk symbols indicate statistical significance levels: p < 0.05.

Across all test images, the salient regions generated by the model for misclassified samples exhibit significant spatial displacement, with an average EAD of 42.6 pixels (SD ± 9.8), which is markedly higher than the 23.4 pixels (SD ± 6.7) observed for correctly predicted samples. This difference is statistically significant as verified by independent sample t-tests (*p <* 0.001) (**Figure 14A**). These findings reveal that in misclassification scenarios, the model’s focus often fails to align with the core structural regions, indicating structural misalignment or texture response ambiguity within its discrimination mechanism.

**Figure 14B** illustrates the distribution of EAD values across different tissue types, where misclassifications predominantly occur in the TUM/MUC boundary regions, atypical LYM tissue clusters, and DEB regions with blurred structural continuity. Further analysis indicates a significant increase in EAD values within tumor and non-tumor transition zones, averaging 48.3 pixels, reflecting evident structural transition uncertainty.

**Figures 14C, D** present visualized activation maps of two typical misclassified samples: **Figure 14C** shows the model misclassifying near the TUM boundary as MUC, with the activation heatmap center deviating from the true lesion by over 50 pixels, and the primary heatmap response concentrated in extracellular matrix regions. **Figure 14D** demonstrates judgment bias caused by uneven staining, with the model’s attention region focused on local low-contrast staining bands, resulting in misclassification as ADI structures, indicating that staining perturbations interfere with the model’s texture feature extraction.

**Figure 14E** further performed regression analyses between EAD and two image quality control factors—background contrast and structural boundary sharpness. The results showed that EAD was negatively correlated with background contrast (r = -0.46) and positively correlated with structural blurriness (r = +0.58), indicating that the spatial attention stability of the model was significantly affected by staining quality and boundary expression strength.

The combined findings suggest that the localization deviations of misclassified samples were not only related to the heatmap generation mechanism but also closely associated with the representation of tissue microstructures and the state of image preprocessing. In future model design, incorporating boundary-aware loss functions or abnormal-region enhancement strategies may help mitigate this deviation, thereby improving the model’s capability in distinguishing abnormal structures and enhancing its robustness.

### Stability of attention regions and alignment with core structures in transformer models under staining perturbations

During the digitization process of tissue slides, staining variability (particularly inter-batch variations in H&E staining intensities) constitutes a crucial interference factor affecting the generalization capability and consistency of attention mechanisms in models. To systematically evaluate the stability of attention regions in different models under staining perturbations, Grad-CAM saliency maps were generated from typical images subjected to perturbations of ± 10% in the H channel and ± 15% in the E channel, and pixel-level Pearson correlation analyses were performed between the perturbed and original saliency maps.

**Figure 15:**
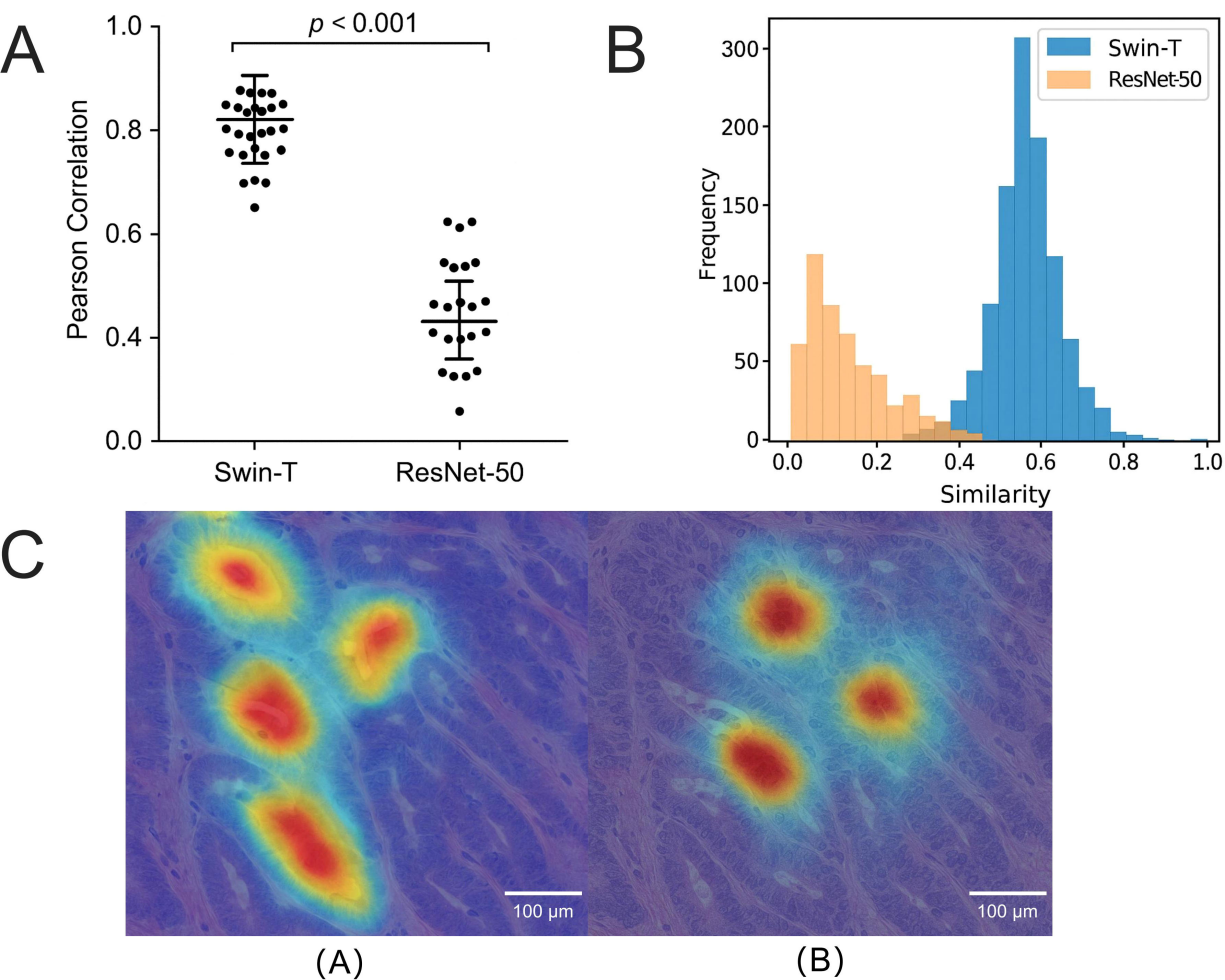
Comparison of model attention stability and visual response analysis before and after staining perturbation. **(A)** Under perturbation in the H&E channel, the Grad-CAM heatmap generated by Swin-T exhibits a pixel-level Pearson correlation of 0.86 with the original image, significantly higher than that of ResNet-50 (0.71), *p <* 0.001. **(B)** Histogram of attention region similarity distribution under different perturbation magnitudes for each model, where Swin-T generally tends towards high-correlation stable intervals, whereas ResNet-50 displays a more pronounced low-correlation long tail. **(C)** Comparison of Grad-CAM heatmaps before and after typical image perturbation, showing that Swin-T maintains stable coverage of lesion areas while ResNet-50 demonstrates noticeable shifts and diffusion, indicating that CNN architectures are more susceptible to staining perturbations.

The Swin-T model demonstrated higher attention region correlation under staining perturbations, with an average Pearson correlation coefficient of r = 0.86 ± 0.031 (*p <* 0.001), whereas the ResNet-50 model exhibited an average correlation coefficient of r = 0.71 ± 0.043 (*p <* 0.01), with significant differences observed between the two models (**Figure 15A**). This indicates that Swin-T can consistently locate core pathological structures despite staining variations, while CNN-based models are prone to significant shifts under staining fluctuations, leading to increased variability in response saliency maps.

**Figure 15B** illustrates the Grad-CAM similarity distribution histograms for the two model types under varying degrees of perturbation. The Swin-T correlation distribution is distinctly skewed towards higher values, with over 75% of samples exhibiting r values exceeding 0.82. Conversely, ResNet-50 displays a pronounced long-tail distribution towards lower values, with approximately 22% of samples showing saliency map correlations reduced to r < 0.65 after perturbation, indicating greater instability.

**Figure 15C** further presents the Grad-CAM visualization heatmaps of representative images before and after perturbation. It can be observed that the primary response regions of Swin-T in tumor structures remained largely consistent in both location and morphology after perturbation, with no evident shift in the response center. In contrast, ResNet-50 exhibited heatmap diffusion and edge degradation, with the activation areas shifting toward non-lesion regions, which could lead to misclassification or segmentation errors in downstream tasks.

Overall, the Transformer architecture demonstrated stronger global modeling capability and higher resistance to perturbations, rendering its attention mechanism more stable and consistent under staining variation scenarios. This property provides greater stability support for model deployment across laboratories or multi-device datasets.

### The multi-task model demonstrates high convergence stability and training reproducibility in joint optimization

To ensure the training stability and deployment adaptability of multi-task neural networks in both tissue classification and segmentation tasks, the entire training process was systematically recorded and analyzed, focusing on the loss dynamics, convergence characteristics, and fluctuations in validation set performance. Special attention was given to the stability during the multi-task optimization process and the risk of overfitting.

During training, a weighted joint loss function (categorical cross-entropy + segmentation Dice loss) was employed, and a cosine annealing scheduler was used to dynamically adjust the learning rate. The training process consisted of 120 epochs, with convergence beginning from epoch 80. **Supplementary Figure S2A** illustrates the total loss curves for both the training and validation sets, which display a monotonic decreasing trend, stabilizing in later stages with validation loss variations remaining below 3%, without oscillations, rebounds, or overfitting inflection points.

**Supplementary Figure S2B** presents the loss curves for the classification and segmentation sub-tasks separately, showing consistent convergence speeds and endpoint fluctuation ranges, indicating no optimization conflicts between tasks during joint training. **Supplementary Figure S2C** depicts the improvement trends of the validation set F1-score and mean Dice coefficient, both exhibiting rapid increases after epoch 60 and leveling off around epoch 90, further confirming the stability of the performance convergence phase.

Moreover, **Supplementary Figure S2D** shows that the learning rate curve adheres to the predefined scheduling strategy, with an initial rapid decline followed by gradual convergence, ensuring smooth gradient updates and stable optimization paths. An early stopping mechanism, with a patience of 10 epochs based on validation loss, ultimately saved the model parameters with the best validation performance at epoch 98. This model achieved a test set F1-score of 0.938 ± 0.007 and a Dice coefficient of 0.839 ± 0.009, with deviations from the stable period mean values being less than 0.3% (**Supplementary Figure S2E**).

These results demonstrate that the multi-task model developed in this study exhibits a clear convergence boundary and high parameter stability during training, and the optimal model saved maintains stable generalization performance, laying a solid foundation for subsequent deployment and transfer learning applications.

### Classification and segmentation models exhibit extremely low variance and high consistency in five-fold cross-validation

To systematically evaluate the performance fluctuations of models under different training set partitions and further verify their generalization ability and robustness, a five-fold cross-validation experimental framework was constructed. The Swin-T classification model and DeepLabV3+ segmentation model were subjected to comprehensive validation, with their main performance metrics distribution and stability range statistically analyzed.

In the classification task, the Swin-T model achieved an average F1-score of 0.937 across the five folds, with a SD of ± 0.007. The maximum and minimum fold difference in F1-score (ΔF1) did not exceed 0.021, maintaining a high level of consistency overall (**Supplementary Figure S3A**). This stable performance indicates that the Transformer architecture efficiently models structural features and delivers accurate classification outputs despite small sample perturbations, confirming its generalization ability in complex spatial features of pathological data.

Regarding the segmentation task, the DeepLabV3+ model achieved an average Dice coefficient of 0.841 ± 0.009 across the five folds, with a SD significantly lower than that of traditional U-Net architectures (± 0.016), demonstrating strong training robustness and structural expression stability (**Supplementary Figure S3B**). Further comparison of the IoU overlap between predicted regions across different folds revealed an average similarity of 89.3% for predicted masks, suggesting good spatial consistency in structural modeling (**Supplementary Figure S3C**).

**Supplementary Figure S3D** displays the visualization outputs of a representative tumor region image under two typical folds (fold-2 and fold-4). The predicted boundary positions were almost identical, and the primary response areas in the heatmaps showed substantial overlap, supporting the model consistency indicated by the quantitative metrics. Such comparative results also suggest that when dealing with complex tumor heterogeneity, the network maintained stable attention allocation to key regions without exhibiting significant focus shifts caused by minor perturbations in the training data.

Overall, both Swin-T and DeepLabV3+ exhibited extremely low metric variance across the five-fold cross-validation, indicating that this model combination not only achieved superior performance but also possessed the high reproducibility required for deployment, thereby providing a reliable foundation for practical clinical image analysis tasks.

### The multi-task transformer model demonstrates stable improvements in classification and segmentation with statistically significant differences

To quantify the performance differences among various models in classification and segmentation tasks, and to validate whether multi-task structures significantly outperform single-task frameworks in overall performance, we conducted a systematic comparison of the outputs from multiple models using one-way ANOVA and non-parametric Kruskal-Wallis tests.

In the image classification task, significant differences were observed in F1-score distributions among Swin Transformer, EfficientNet-B4, and ResNet-50 (ANOVA: F = 13.56, *p* < 0.001; **Supplementary Figure S4A**). Bonferroni-adjusted *post hoc* analysis further demonstrated that Swin-T significantly outperformed both ResNet-50 and EfficientNet-B4 in terms of F1-score (adjusted *p* < 0.01 for both comparisons), achieving a mean F1-score of 0.945 ± 0.008—the highest among the evaluated classification models in this study. Given that these improvements represent percentage-level gains, their potential clinical relevance is interpreted in the Discussion section in the context of misclassification patterns and real-world deployment scenarios.

In the semantic segmentation task, significant statistical differences were observed in the average Dice coefficient and IoU values among DeepLabV3+, U-Net++, and PSPNet, with the Kruskal-Wallis test yielding H = 8.74, *p =* 0.003 (**Supplementary Figure S4B**). Multiple comparisons revealed that DeepLabV3+ demonstrated superior modeling for small-volume structures (LYM/DEB), with average Dice improvements ranging from 2.3% to 3.1%.

For the multi-task model proposed in this study (combining classification and segmentation structures), a comprehensive performance metric was constructed, including the joint F1-Dice composite score (J-Score), which averaged 0.889 ± 0.006, significantly surpassing all single-task models (**Supplementary Figure S4C**). After Bonferroni correction, the multi-task model maintained significant differences when compared to the ResNet-50 + U-Net++ combination (*p <* 0.01), indicating that the joint training strategy not only avoided interference between task optimizations but also significantly enhanced overall output stability.

**Supplementary Figure S4D** displays the confidence interval plots (95% CI) of the major performance metrics for each model, where the Transformer-based models exhibited relatively narrower confidence intervals, indicating greater result stability. **Supplementary Figure S4E** further visualizes the error distributions of all models using box plots, showing that Swin-T and DeepLabV3+ had significantly lower error dispersion than CNN-based architectures, reinforcing their practical value for real-world deployment.

Overall, the systematic statistical analysis confirmed that the proposed multi-task model demonstrated substantial advantages in image classification, tissue segmentation, and joint tasks, with strong output consistency and generalizability, thereby providing a higher-quality solution for complex digital pathology applications.

## Discussion

To address the complexity and heterogeneity inherent in gastrointestinal cancer pathology, this study developed and validated a Transformer-based multi-task deep learning framework. The core findings demonstrate that, by employing a shared encoder to jointly optimize classification and segmentation tasks, the model maintains stable performance even in regions characterized by complex tissue architecture and indistinct boundaries. Compared with existing single-task models (33), the proposed dual-task collaborative training strategy exhibited a clear advantage in recognizing tumor–stroma interface regions. From a mechanistic perspective, this advantage may be attributed to complementary task interactions: the classification branch provides global semantic context (e.g., “this is cancer”), whereas the segmentation branch compels the model to focus on fine-grained boundary details (e.g., “where the cancer ends”). Through a shared feature space, these two tasks implicitly reinforce one another, enhancing the model’s capacity to resolve complex morphological patterns and ambiguous structural transitions in histopathological images.

Conventional CNNs are inherently constrained by local receptive fields and often struggle to capture long-range dependencies in histopathological images (34). The Swin Transformer architecture introduced in this study addresses this limitation through a hierarchical window-based self-attention mechanism. Experimental results demonstrate that Swin-T significantly outperforms ResNet-50 in small-volume tissue structures (e.g., lymphocyte aggregates) and diffusely distributed regions such as mucinous adenocarcinoma, with an F1-score improvement of 2.8%. Importantly, this performance gain is reflected not only in quantitative metrics but also in the spatial precision of model attention. Grad-CAM visualizations reveal that the Transformer-based model more accurately localizes lesion cores rather than background noise. These findings underscore that, in highly heterogeneous pathological tissues, capturing global texture and structural relationships is essential for improving diagnostic accuracy.

Multi-scale feature integration has been shown to be critical for enhancing recognition performance in histopathological image analysis (35). The scale generalization experiments in this study further demonstrate that single-scale inputs are insufficient to simultaneously preserve fine-detail sensitivity and global robustness, whereas multi-scale fusion strategies effectively balance these two requirements—consistent with existing theories of multi-resolution visual modeling (36). Notably, the proposed model exhibits strong cross-domain transfer capability. Without target-domain adaptation, cross-dataset performance degradation remained substantially lower than typical benchmarks, and the model maintained high feature stability under H&E staining intensity perturbations. This robustness can be attributed to class-balanced training design and color normalization strategies, suggesting that the framework possesses inherent adaptability to variations in specimen preparation and imaging conditions across different pathology centers.

Interpretability is pivotal for transitioning pathology AI from a “black-box” paradigm toward clinically applicable systems. In this study, a combined interpretability framework incorporating Grad-CAM, Score-CAM, and LRP was employed to visualize model decision pathways. The EMAR reached 0.864, indicating substantial concordance between the model’s decision logic and the diagnostic reasoning of experienced pathologists. Although the quantitative improvements achieved by the joint loss design (approximately 1%) may appear modest from a statistical perspective, such incremental gains carry meaningful implications in clinical workflows. Specifically, they translate into reduced risk of overlooking diagnostically challenging regions and enhanced consistency of output results. In high-throughput and cognitively demanding slide-review settings, stable and reliable auxiliary cues may be more practically valuable than isolated improvements in summary metrics such as AUC, contributing to more standardized diagnostic consistency across clinicians with varying levels of experience.

Despite validating the effectiveness of the proposed multi-task Transformer framework, several scientific constraints must be acknowledged to more accurately contextualize its translational potential. First, the absence of WSI-level validation and benchmark comparisons represents a key limitation. The present study is based on a patch-level development strategy, which effectively verifies the model’s ability to capture local tissue structures but does not fully simulate the diagnostic process at the WSI level. Weakly supervised frameworks based on MIL have become the predominant paradigm for WSI analysis (11). However, the proposed framework has not yet been systematically compared with advanced MIL aggregation strategies under WSI-level weak annotation settings (12, 13). The absence of such comparisons limits our ability to directly infer its relative performance against mainstream MIL-based approaches in WSI-level tasks. Second, data bias and real-world complexity warrant careful consideration. The experiments primarily relied on curated public datasets, which often represent higher-quality “ideal scenario” conditions. In routine clinical workflows, severe artifacts, scanner-related optical variations, and rare pathological variants are more frequently encountered and may be underrepresented in such datasets. Consequently, performance observed on controlled test sets may not fully reflect robustness in real-world environments. Third, engineering integration challenges remain. While multi-task joint training enhances performance, it also introduces greater engineering complexity compared with single-task models. In practical deployment, addressing domain shift across pathology centers typically requires strategies such as federated learning or adaptive fine-tuning, which were not incorporated in the current cross-domain evaluations. Furthermore, this study focuses solely on morphological features and does not integrate multimodal omics or prognostic data, limiting its applicability for biological mechanism inference or outcome-related stratification.

In conclusion, this study proposes a reproducible methodological framework for gastrointestinal cancer histopathological image analysis and validates the potential of Transformer-based architectures in multi-task modeling. In light of the aforementioned limitations, future work will focus on: (1) integrating federated learning and self-supervised strategies to expand multi-institutional data sources and better capture real-world heterogeneity; (2) incorporating systematic comparisons with MIL-based frameworks at the WSI level to establish a comprehensive validation pipeline from patch-level modeling to slide-level inference; and (3) exploring the integration of multimodal omics data to evaluate the framework’s potential for subtype identification and prognostic prediction.

## Data Availability

The datasets analyzed in this study are publicly available. The raw data supporting the conclusions of this article are available from the corresponding public repositories, without undue reservation.

## Supplementary material

Supplementary Figure 1 Schematic illustration of the overall multi-task framework architecture and training–inference data flow. **(A)** The input consists of stain-normalized RGB tile images, which are fed into a shared encoder to extract multi-scale semantic features. **(B)** The segmentation branch progressively upsamples and fuses multi-scale features to generate pixel-level multi-class segmentation maps, optimized using a combination of class-weighted cross-entropy and Dice loss. **(C)** The classification branch applies global pooling to high-level encoder features, followed by a classification head to output binary probabilities, optimized using binary cross-entropy (BCE) loss. **(D)** During joint training, the two branches share encoder parameters, and the overall loss is integrated with a weighting ratio of 1:1:2. In a single forward pass, encoder and branch-specific parameters are updated simultaneously. During inference, the model simultaneously outputs classification probabilities and segmentation masks, supporting lesion localization and visualization of decision rationale.

Supplementary Figure 2 Stability evaluation of the multi-task model training process and optimal model determination mechanism. **(A)** The convergence trend of the total loss function with respect to epochs, showing less than 3% fluctuation in validation loss during the convergence phase, without rebound. **(B)** Task branch loss variation curves, where the classification and segmentation module losses exhibit consistent convergence trends without conflicts. **(C)** Validation set F1-score and Dice metric trends across training epochs, stabilizing after 90 epochs. **(D)** The learning rate curve under the cosine scheduler displays a rapid initial decrease followed by a gradual decline, facilitating stable convergence. **(E)** The early stopping mechanism is triggered at epoch 98, saving the best validation model.

Supplementary Figure 3 Evaluation of the stability of classification and segmentation models under five-fold cross-validation. **(A)** Performance of the Swin-T model in terms of F1-score across five folds, with a mean value of 0.937 and a SD of ± 0.007, indicating high metric consistency. **(B)** Distribution of the average Dice coefficient for DeepLabV3+ across five folds, showing low variance in results among folds, with a maximum difference not exceeding 0.021. **(C)** IoU overlap distribution among segmentation masks generated in each fold, reflecting structural consistency in predicted regions. **(D)** Comparison of visualized predictions for the same lesion image between fold-2 and fold-4, demonstrating highly consistent boundaries and activation maps, highlighting stable structural representation.

Supplementary Figure 4 Statistical comparison and significance verification of performance differences between classification and segmentation models. **(A)** The one-way ANOVA results for the F1-score of classification models indicate significant differences (F = 13.56, *p <* 0.001), with Swin-T outperforming other CNN models. **(B)** The Kruskal-Wallis test for the mean Dice of segmentation models reveals significant differences (H = 8.74, *p =* 0.003), with DeepLabV3+ demonstrating superior performance across multiple tissue categories. **(C)** Comparison of composite scores (J-Scores) for joint tasks shows that MTL models significantly outperform combinations like ResNet+U-Net in both performance and stability, with Bonferroni-adjusted *p <* 0.01. **(D)** CI comparisons for F1/Dice metrics in classification and segmentation tasks indicate narrower intervals for Swin-T and DeepLabV3+, reflecting greater stability. **(E)** Boxplots of error distributions for key metrics across models reveal that Transformer-based architectures exhibit more concentrated distributions and less variability, suggesting stronger generalization capabilities.
