# Supplementary figures and images for: A multi-task and explainable swin transformer framework for cross-scale computational pathology in gastrointestinal cancer
Source: Front Oncol. 2026 Apr 21;16:1749675. doi: 10.3389/fonc.2026.1749675 (PMC13138889; doi:10.3389/fonc.2026.1749675)

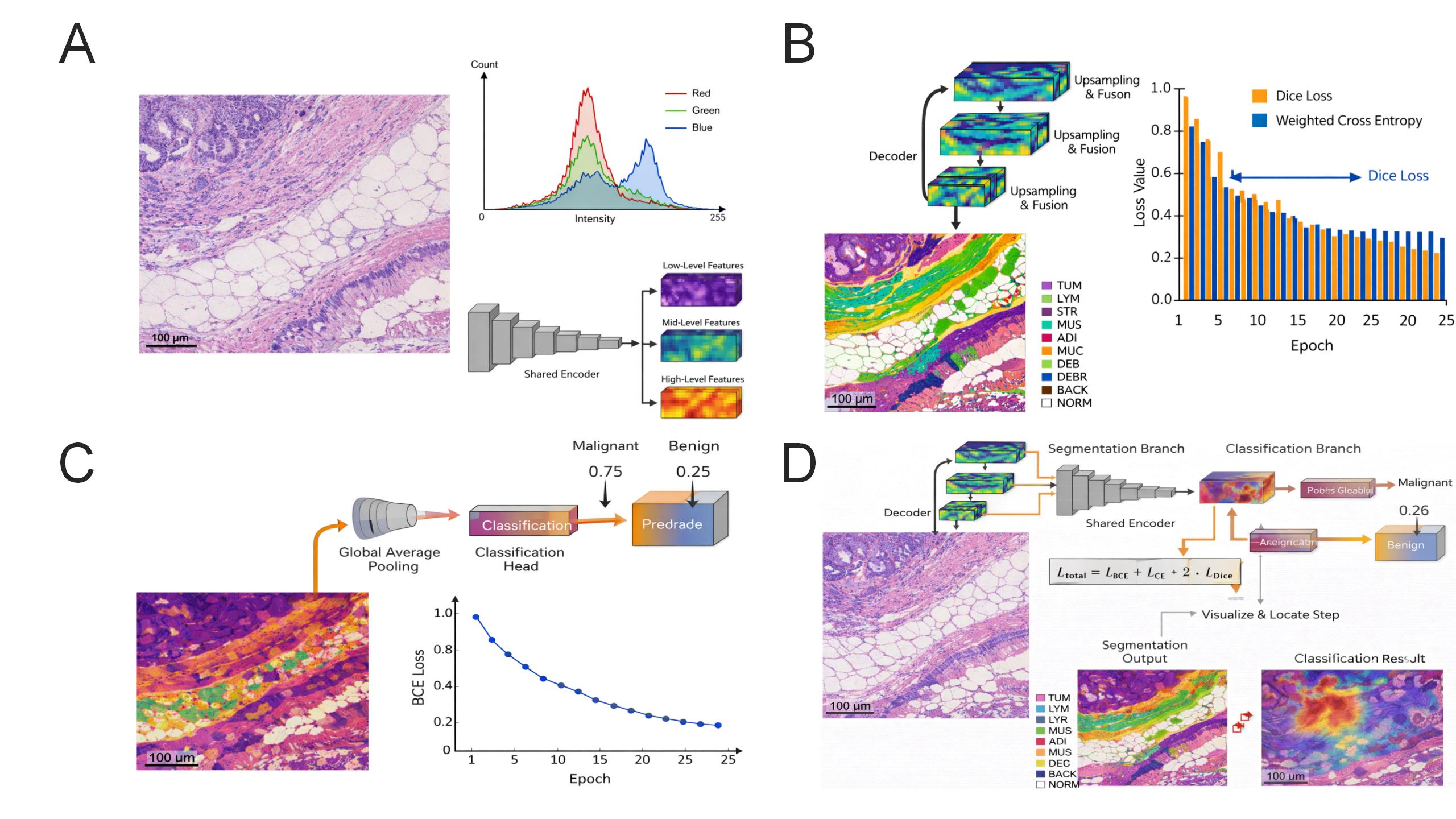

Supplement: Supplementary Figure 1 — Schematic illustration of the overall multi-task framework architecture and training–inference data flow. (A) The input consists of stain-normalized RGB tile images, which are fed into a shared encoder to extract multi-scale semantic features. (B) The segmentation branch progressively upsamples and fuses multi-scale features to generate pixel-level multi-class segmentation maps, optimized using a combination of class-weighted cross-entropy and Dice loss. (C) The classification branch applies global pooling to high-level encoder features, followed by a classification head to output binary probabilities, optimized using binary cross-entropy (BCE) loss. (D) During joint training, the two branches share encoder parameters, and the overall loss is integrated with a weighting ratio of 1:1:2. In a single forward pass, encoder and branch-specific parameters are updated simultaneously. During inference, the model simultaneously outputs classification probabilities and segmentation masks, supporting lesion localization and visualization of decision rationale. [file Image1.jpeg]

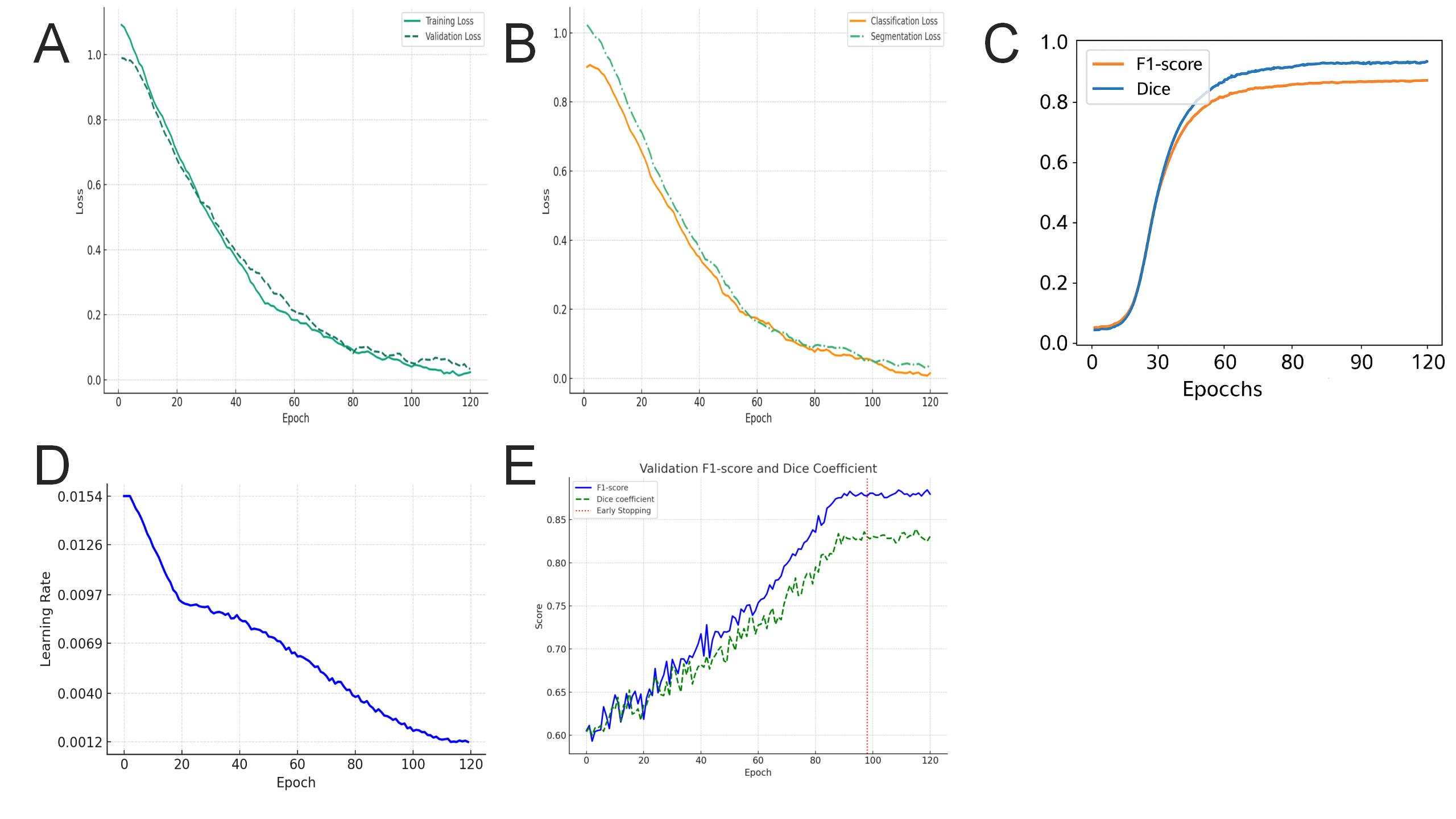

Supplement: Supplementary Figure 2 — Stability evaluation of the multi-task model training process and optimal model determination mechanism. (A) The convergence trend of the total loss function with respect to epochs, showing less than 3% fluctuation in validation loss during the convergence phase, without rebound. (B) Task branch loss variation curves, where the classification and segmentation module losses exhibit consistent convergence trends without conflicts. (C) Validation set F1-score and Dice metric trends across training epochs, stabilizing after 90 epochs. (D) The learning rate curve under the cosine scheduler displays a rapid initial decrease followed by a gradual decline, facilitating stable convergence. (E) The early stopping mechanism is triggered at epoch 98, saving the best validation model. [file Image2.jpeg]

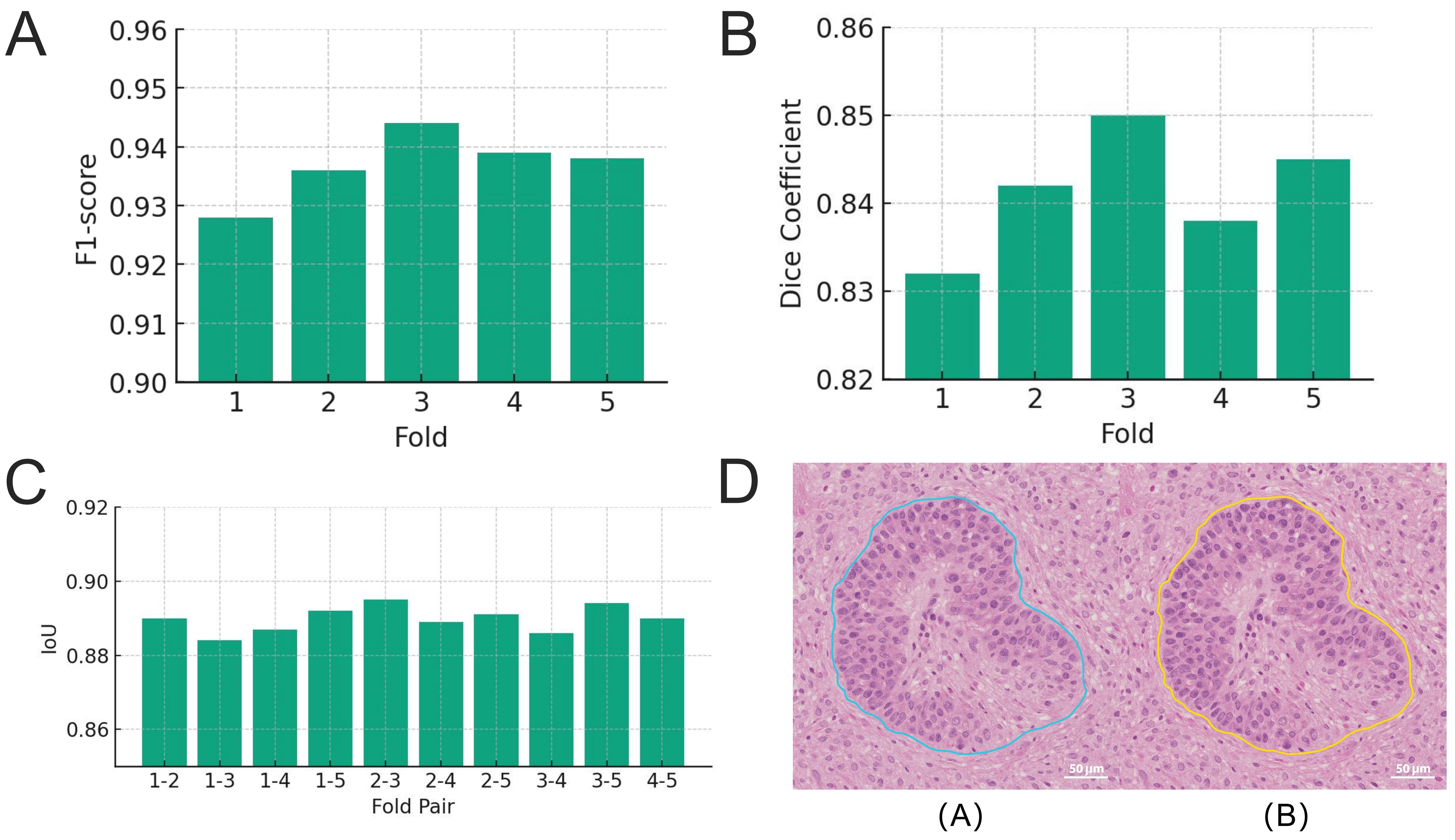

Supplement: Supplementary Figure 3 — Evaluation of the stability of classification and segmentation models under five-fold cross-validation. (A) Performance of the Swin-T model in terms of F1-score across five folds, with a mean value of 0.937 and a SD of ± 0.007, indicating high metric consistency. (B) Distribution of the average Dice coefficient for DeepLabV3+ across five folds, showing low variance in results among folds, with a maximum difference not exceeding 0.021. (C) IoU overlap distribution among segmentation masks generated in each fold, reflecting structural consistency in predicted regions. (D) Comparison of visualized predictions for the same lesion image between fold-2 and fold-4, demonstrating highly consistent boundaries and activation maps, highlighting stable structural representation. [file Image3.jpeg]

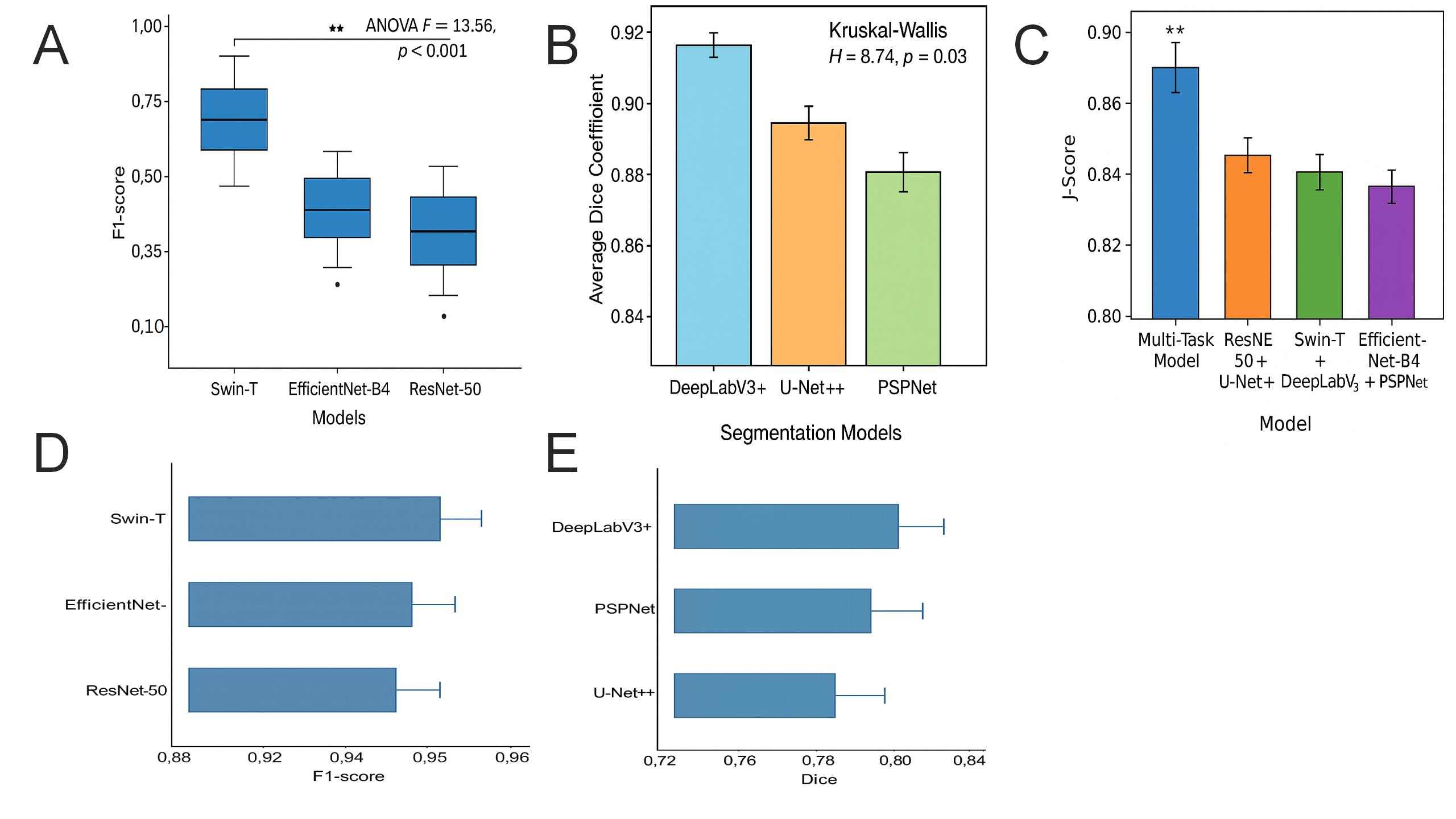

Supplement: Supplementary Figure 4 — Statistical comparison and significance verification of performance differences between classification and segmentation models. (A) The one-way ANOVA results for the F1-score of classification models indicate significant differences (F = 13.56, p < 0.001), with Swin-T outperforming other CNN models. (B) The Kruskal-Wallis test for the mean Dice of segmentation models reveals significant differences (H = 8.74, p = 0.003), with DeepLabV3+ demonstrating superior performance across multiple tissue categories. (C) Comparison of composite scores (J-Scores) for joint tasks shows that MTL models significantly outperform combinations like ResNet+U-Net in both performance and stability, with Bonferroni-adjusted p < 0.01. (D) CI comparisons for F1/Dice metrics in classification and segmentation tasks indicate narrower intervals for Swin-T and DeepLabV3+, reflecting greater stability. (E) Boxplots of error distributions for key metrics across models reveal that Transformer-based architectures exhibit more concentrated distributions and less variability, suggesting stronger generalization capabilities. [file Image4.jpeg]
