# Supplementary material for: A multi-task and explainable swin transformer framework for cross-scale computational pathology in gastrointestinal cancer
Source: Front Oncol. 2026 Apr 21;16:1749675. doi: 10.3389/fonc.2026.1749675 (PMC13138889; doi:10.3389/fonc.2026.1749675)
Supplement: Supplementary file 5 [file Table1.docx]

**Table S1. Summary of quality control filtering across datasets.**

| **Dataset** | **Original Image Count** | **BRISQUE Excluded(n, %)** | **Low Tissue Area Excluded(n, %)** | **Final Retained Images** |
| --- | --- | --- | --- | --- |
| GasHisSDB | 90000 | 4350 (4.8%) | 1430(1.6%) | 84220 |
| GCHTID | 16000 | 430 (2.7%) | 520(3.3%) | 15050 |
| Total | 106000 | 4780 (4.5%) | 1950(1.8%) | 99270 |

Note: BRISQUE score threshold > 50 was applied to exclude low-quality images (blurred, over-smoothed edges, distorted colors). Low-tissue slices were excluded using OpenCV Canny edge detection with an area-threshold rule (tissue area < 40%). The overall exclusion rate was controlled within 10%.
