# Supplementary material for: A multi-task and explainable swin transformer framework for cross-scale computational pathology in gastrointestinal cancer
Source: Front Oncol. 2026 Apr 21;16:1749675. doi: 10.3389/fonc.2026.1749675 (PMC13138889; doi:10.3389/fonc.2026.1749675)
Supplement: Supplementary file 6 [file Table2.docx]

**Table S2. Summary of label verification and stratified sampling balance (GCHTID)**

| **Process** | **Metric** | **Value** | **Notes** |
| --- | --- | --- | --- |
| Label verification (training set) | Sampling fraction | 5% | Random sample from the training set |
| Label verification (training set) | Reviewed sample size | 1,555 images | Double-blinded review by two pathologists |
| Label verification (training set) | Inter-rater agreement | Cohen’s Kappa = 0.82 (95% CI 0.79–0.85) | Overall agreement was strong |
| Label verification (training set) | Images with notable label discrepancy | 88 (5.7% of reviewed sample) | Resolved by rule-based adjudication |
| Label verification (training set) | Post-review action | Relabeled: 64; Removed: 24 | To mitigate label noise |
| Stratified split balance | Target per class | 2,000 ± 15 images | 8 tissue classes |
| Stratified split balance | Range across classes | 1,985–2,013 | Counts in the training set |
| Stratified split balance | SD of class counts | 9.1 | Lower indicates better balance |
| Stratified split balance | Kruskal-Wallis test across subsets | H = 2.03; p = 0.91 | No significant difference among train/val/test |
| Stratified split balance | Max subset proportion deviation | ±0.7% (DEB) | Most classes were within ±0.5% |

Note: Table S2 summarizes the statistics that were moved from the main Results to the Supplementary Material, including (i) label verification on a random 5% sample from the training set and (ii) balance assessment of the stratified train/validation/test split for the 8 tissue classes. Inter-rater agreement was quantified using Cohen’s Kappa with the corresponding 95% confidence interval (CI). Images with notable label discrepancy were resolved by predefined adjudication rules and were either relabeled or removed to mitigate label noise. Distribution balance across subsets was evaluated using the Kruskal–Wallis test on per-class counts; a two-sided p value < 0.05 was considered statistically significant. SD denotes standard deviation. DEB denotes the maximum absolute deviation of subset class proportions from the expected proportion under stratified sampling.
