# Supplementary material for: A multi-task and explainable swin transformer framework for cross-scale computational pathology in gastrointestinal cancer
Source: Front Oncol. 2026 Apr 21;16:1749675. doi: 10.3389/fonc.2026.1749675 (PMC13138889; doi:10.3389/fonc.2026.1749675)
Supplement: Supplementary file 7 [file Table3.docx]

**Table S3. Performance comparison between the proposed method and baseline methods.**

| **Task** | **Dataset** | **Model** | **Accuracy** | **AUC** | **F1-score** | **Mean IoU** | **Mean Dice** |
| --- | --- | --- | --- | --- | --- | --- | --- |
| Tumor classification (binary) | GasHisSDB (160×160) | ResNet-50 | 0.919 | 0.907 | 0.917 ± 0.010 | — | — |
| Tumor classification (binary) | GasHisSDB (160×160) | EfficientNet-B4 | 0.936 | 0.929 | 0.933 ± 0.009 | — | — |
| Tumor classification (binary) | GasHisSDB (160×160) | Swin Transformer (Swin-T) | 0.948 | 0.965 | 0.945 ± 0.008 | — | — |
| Tissue segmentation (multi-class) | GCHTID (224×224) | U-Net++ | — | — | — | 0.768 ± 0.013 | 0.824 ± 0.012 |
| Tissue segmentation (multi-class) | GCHTID (224×224) | DeepLabV3+ (ResNet101 backbone) | — | — | — | 0.792 ± 0.012 | 0.847 ± 0.011 |
| Joint classification + segmentation (multi-task) | GCHTID (224×224) | Proposed multi-task model (R2U-Net) | — | — | 0.938 ± 0.007 | — | 0.839 ± 0.009 |

Note: All values are reported as mean ± standard deviation where applicable. “—” indicates that the metric is not applicable or not reported for that task in the manuscript.
